# Comparative efficacy and outcomes of different treatments for pathologically high-risk superficial esophageal cancer: a systematic review and network meta-analysis

**DOI:** 10.3389/fonc.2026.1866820

**Published:** 2026-07-23

**Authors:** Hongzhi He, Hanlu Zhang, Haowen Lv, Ningying Ding, Wenping Wang

**Affiliations:** 1 Department of Thoracic Surgery, West China Hospital, Sichuan University, Chengdu, China; 2 West China School of Medicine, Sichuan University, Chengdu, China; 3 Operating Room, Department of Anesthesiology, West China Hospital, West China School of Nursing, Sichuan University, Chengdu, Sichuan, China

**Keywords:** adjuvant therapy, chemoradiotherapy, endoscopic resection, esophagectomy, network meta-analysis, non-curative resection, superficial esophageal cancer

## Abstract

**Background:**

Endoscopic resection (ER) is increasingly used for superficial esophageal cancer (SEC), but pathological examination may reveal features associated with lymph-node metastasis or recurrence. The optimal management of patients with pathologically high-risk SEC remains uncertain because high-quality randomized evidence is lacking and direct comparisons among the available treatment strategies remain limited.

**Methods:**

We performed a systematic review and frequentist network meta-analysis according to PRISMA (PROSPERO CRD420251164650). PubMed, Embase, Web of Science, and the Cochrane Library were searched from inception to December 17, 2025. Random-effects models were used as the primary analyses for overall survival (OS), recurrence-free survival (RFS), and disease-specific survival (DSS). Study-level subgroup analysis, network meta-regression, and sensitivity analyses were used to examine the robustness and comparability of the evidence.

**Results:**

Thirty-four studies were included. The OS, RFS, and DSS networks contained 2,621, 2,173, and 1,274 participants, respectively. Compared with primary esophagectomy (ESO), ER followed by esophagectomy (ER+ESO) had an OS hazard ratio (HR) of 0.562 (95% CI 0.311-1.015; P = 0.056). The corresponding HRs were 0.480 for RFS (95% CI 0.235-0.983; P = 0.045) and 0.206 for DSS (95% CI 0.069-0.619; P = 0.005). No other strategy differed significantly from ESO. Global inconsistency was not detected for OS or DSS, whereas the RFS network showed significant between-design inconsistency. Study-level subgroup and network meta-regression analyses did not identify statistically significant effect modification by pathological-stage composition, T1b proportion, or lymphovascular invasion burden.

**Conclusions:**

ER+ESO was associated with favorable RFS and DSS estimates in selected patients suitable for surgery, with a similar direction of effect for OS. These findings may support ER+ESO as an important post-ER treatment option when surgical tolerance is acceptable. ER followed by chemoradiotherapy may represent a clinically relevant organ-preserving strategy, particularly for patients in whom esophagectomy is not preferred or feasible.

**Systematic Review Registration:**

https://www.crd.york.ac.uk/PROSPERO/view/CRD420251164650, identifier CRD420251164650.

## Introduction

Esophageal cancer (EC) is the 11th most common cancer and the seventh leading cause of cancer death worldwide (1). Although the incidence of EC, particularly esophageal squamous cell carcinoma (ESCC), has declined in several traditionally high-risk regions, many patients are still diagnosed with locally advanced disease (2). The five-year survival rate for locally advanced EC remains approximately 10%-30% (3).

Improved screening and endoscopic imaging have increased the detection of early-stage EC (4–6). Five-year survival can exceed 80% in appropriately selected patients with early disease (7–9). Endoscopic mucosal resection or endoscopic submucosal dissection preserve the esophagus and have become standard treatments for superficial lesions with a low risk of lymph-node metastasis (10, 11).

However, a discrepancy between clinical and pathological staging is unavoidable in some patients selected for endoscopic resection (ER). Pathological examination may identify non-curative features, including pT1a disease with lymphovascular invasion (LVI), submucosal invasion, a positive vertical margin, or poor differentiation (12–14). These pathological features are not prognostically equivalent. Both invasion depth and LVI are associated with metastatic risk, while LVI identifies a particularly high-risk subgroup even among tumors with comparable invasion depth (15–17). Nevertheless, in real-world clinical practice, these heterogeneous pathological features are evaluated within the same post-ER decision framework, in which the purpose of additional treatment is to reduce recurrence and metastasis balancing potential benefit against the patient’s ability to tolerate further therapy.

In clinical practice, the optimal management strategy for pathologically high-risk superficial esophageal cancer (SEC) remains uncertain. Direct comparisons are limited, while treatment selection is influenced by age, comorbidity, performance status, pathological risk, institutional practice, and patient preference. Previous pairwise meta-analyses compared surgical and non-surgical additional treatment after non-curative ER, but they could not compare all available sequential treatment pathways within a unified framework (18, 19). Therefore, we conducted a systematic review and network meta-analysis to compare post-ER treatment strategies for patients with pathologically high-risk superficial esophageal cancer.

## Methods

### Search strategy

**Figure 1:**
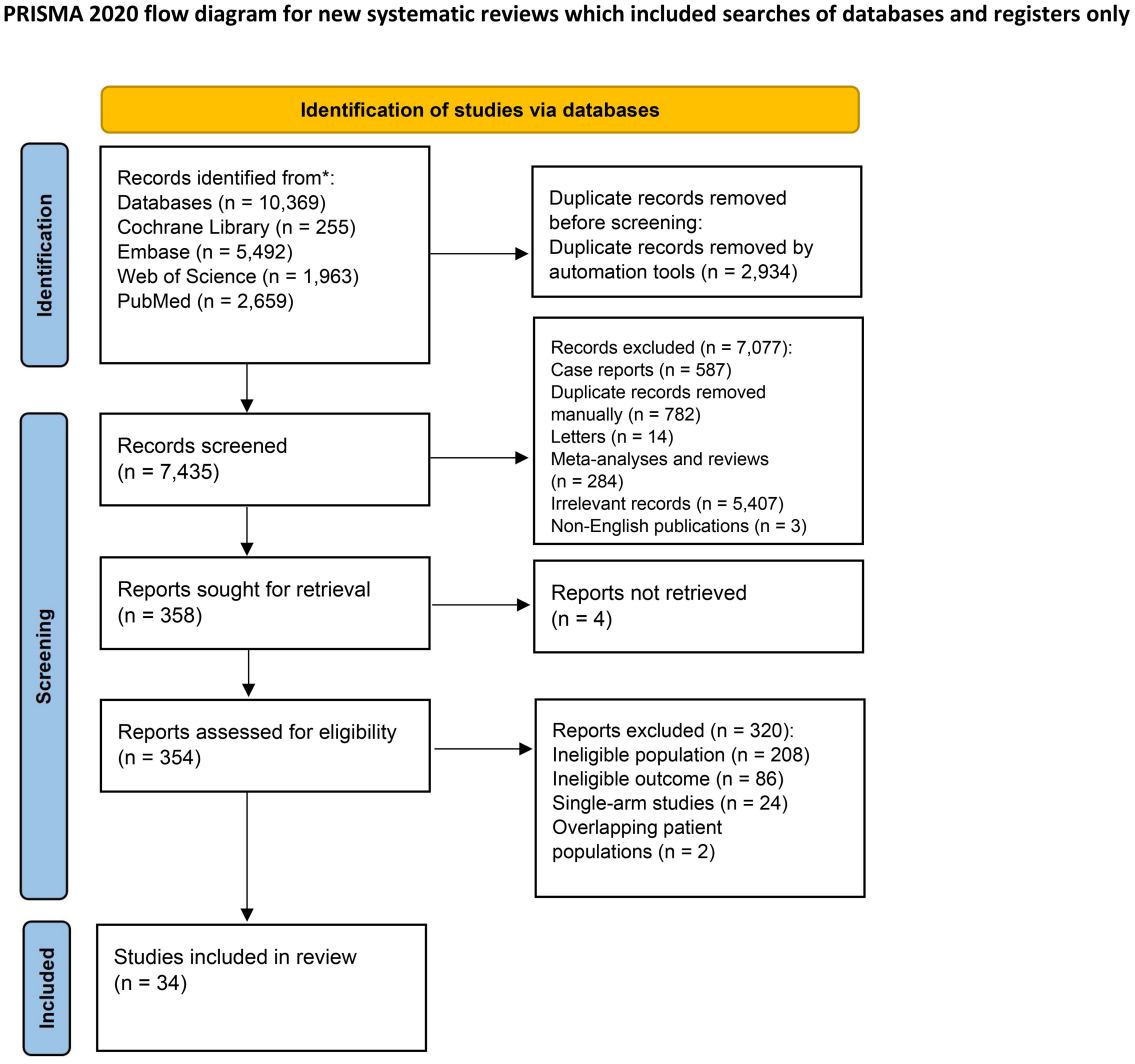
PRISMA study identification and selection flow diagram.

This systematic review and network meta-analysis was registered with PROSPERO (CRD420251164650) and conducted according to the PRISMA 2020 statement (20). PubMed, Embase, Web of Science, and the Cochrane Library were searched from inception to December 17, 2025. The search combined terms for esophageal cancer, esophagectomy, endoscopic resection, chemoradiotherapy, and related treatments. The complete database-specific strategies are provided in **Supplementary Table 1**, and the selection process is shown in **Figure 1**.

### Selection criteria

#### Inclusion criteria

1. Participants: Patients with pathological stage T1 SEC and at least one high-risk feature identified after ER or esophagectomy (ESO) were included. Definitions of non-curative ER varied across studies; eligibility was determined using prespecified pathological high-risk features rather than the terminology used in each publication. These features included pT1a disease with LVI, pT1b submucosal invasion, poor differentiation, or a positive vertical margin.
2. Interventions: ESO; ER followed by surveillance (ER); ER followed by ESO (ER+ESO); ER followed by chemoradiotherapy (ER+CRT); ER followed by radiotherapy (ER+RT); or ER followed by selective adjuvant therapy (ER+SAT). ER+SAT was a study-defined heterogeneous node in which subsequent management could include selective ESO, CRT, RT, chemotherapy, lymph-node dissection, or observation. Study-specific components are reported in **Supplementary Table 2**.
3. Outcomes: Overall survival (OS), recurrence-free survival (RFS), disease-specific survival (DSS), and adverse events (AEs).
4. Study designs: Randomized controlled trials or comparative observational studies, including cohort and case-control studies were included.

#### Exclusion criteria

1. Studies without comparative survival data for at least two eligible treatments. We also excluded studies lacking a reported hazard ratio, a usable Kaplan-Meier curve, or sufficient information to estimate a time-to-event effect.
2. Case reports, conference abstracts, reviews, meta-analyses, and duplicate publications.
3. Reports that used the same effect cohort for the same treatment comparison and outcome. Potentially related reports were assessed using recruitment centers and periods, eligibility criteria, treatment groups, sample sizes, and outcomes. Sharing an institution or recruitment period alone was not considered sufficient evidence of duplication when the analyzed treatment populations differed. When the same effect cohort was reported more than once, the report with the most complete and relevant network data was retained.

### Data extraction and processing

We extracted study design, study period, country, sample size, treatment strategy, follow-up, outcomes, and key patient and tumor characteristics, including age, sex, comorbidity, performance status, histology, tumor size and location, pathological T category, LVI, vertical margin status, and poor differentiation. When multiple summaries were reported, values corresponding to the treatment arms contributing to each network comparison were preferred. Continuous variables were retained as originally reported, and means, medians, lymphatic invasion, and venous invasion were not converted or combined when the required assumptions were uncertain.

The primary outcomes were analyzed as time-to-event data. Reported hazard ratios (HRs) were used when they matched the relevant treatment contrast and outcome cohort. When suitable HRs were unavailable, HRs were reconstructed from published Kaplan–Meier curves using established methods (21, 22); if neither HRs nor usable curves were available, estimates were derived from follow-up, group sizes, and event counts according to Tierney et al. (23). Each estimate was included only when the underlying cohort could be matched to the treatment population used in the network.

For adverse events, we recorded the grading system, denominator, and whether studies reported patients with at least one grade 3 or higher event or category-specific event counts. Because grading systems and reporting units differed across studies, safety outcomes were summarized qualitatively.

### Assessment of risk of bias

Methodological quality was assessed using the Newcastle–Ottawa Scale. Two reviewers (He and Lv) independently assessed each study. Disagreements were resolved through discussion and, when necessary, adjudication by a third reviewer (Zhang). The final consensus scores ranged from 5 to 9, with a mean of 6.7; 10 studies (29.4%) scored at least 8. Detailed assessments are provided in **Supplementary Table 3**.

### Statistical analysis

Statistical analyses were performed in R version 4.6.0 using ‘netmeta’ version 3.6-1 (24). Connected networks were constructed separately for OS, RFS, and DSS, with ESO as the reference treatment. Random-effects inverse-variance models were used as the primary analyses, because clinical and methodological heterogeneity was expected across the predominantly observational evidence base. Between-study variance estimated by restricted maximum likelihood. Common-effect models were examined in sensitivity analyses.

Multi-arm studies contributed all eligible contrasts under a common study to account for within-study correlation. Heterogeneity was assessed using Cochran’s Q, τ², and I². Random-effects P-scores were reported as descriptive ranking measures. Direct estimates in the league tables were obtained using the ‘meta’ package (version 8.5-0).

Transitivity was assessed by comparing age, comorbidity or performance status, pathological T category, LVI, vertical margin status, and poor differentiation across treatment nodes. Local and global inconsistency were evaluated using random-effects node splitting and the design-by-treatment interaction model, respectively. Design-detachment analysis was used when global inconsistency was detected.

Exploratory subgroup analysis compared pT1b-restricted with mixed pT1a/pT1b studies. Common-slope network meta-regression was performed using ‘netmetareg’, with T1b and LVI proportions evaluated separately per 10-percentage-point increase. Lymphatic and venous invasion were combined only when double counting could be excluded. Age was not meta-regressed because summary measures were incompatible. Sensitivity analyses separately excluded ER+SAT, ER+RT, and the identified outcome-specific cohort mismatch, or restricted the evidence to verified propensity-score-matched cohorts and post-ER strategies. Comparison-adjusted funnel plots were examined when feasible. All tests were two-sided, with P<0.05 considered statistically significant, and exploratory analyses were interpreted cautiously.

## Results

After deduplication, 7,435 records were identified. Two reviewers (He and Ding) independently screened titles and abstracts and assessed 354 full-text articles, resulting in 34 included studies (10, 12, 13, 25–55) (**Figure 1**). To avoid double counting, the later 65-centre Japanese pooled study by Yamamoto et al. (56) was excluded because its 2006–2015 cohort was likely to include patients from centers represented in several earlier eligible reports (12, 13, 29–31, 33, 35, 47, 50). The earlier reports were retained because they provided the treatment-specific comparisons and outcome data used to construct the network. The preliminary report by Suzuki et al. (57) was excluded in favor of the larger updated cohort with longer follow-up (46).

**Figure 2:**
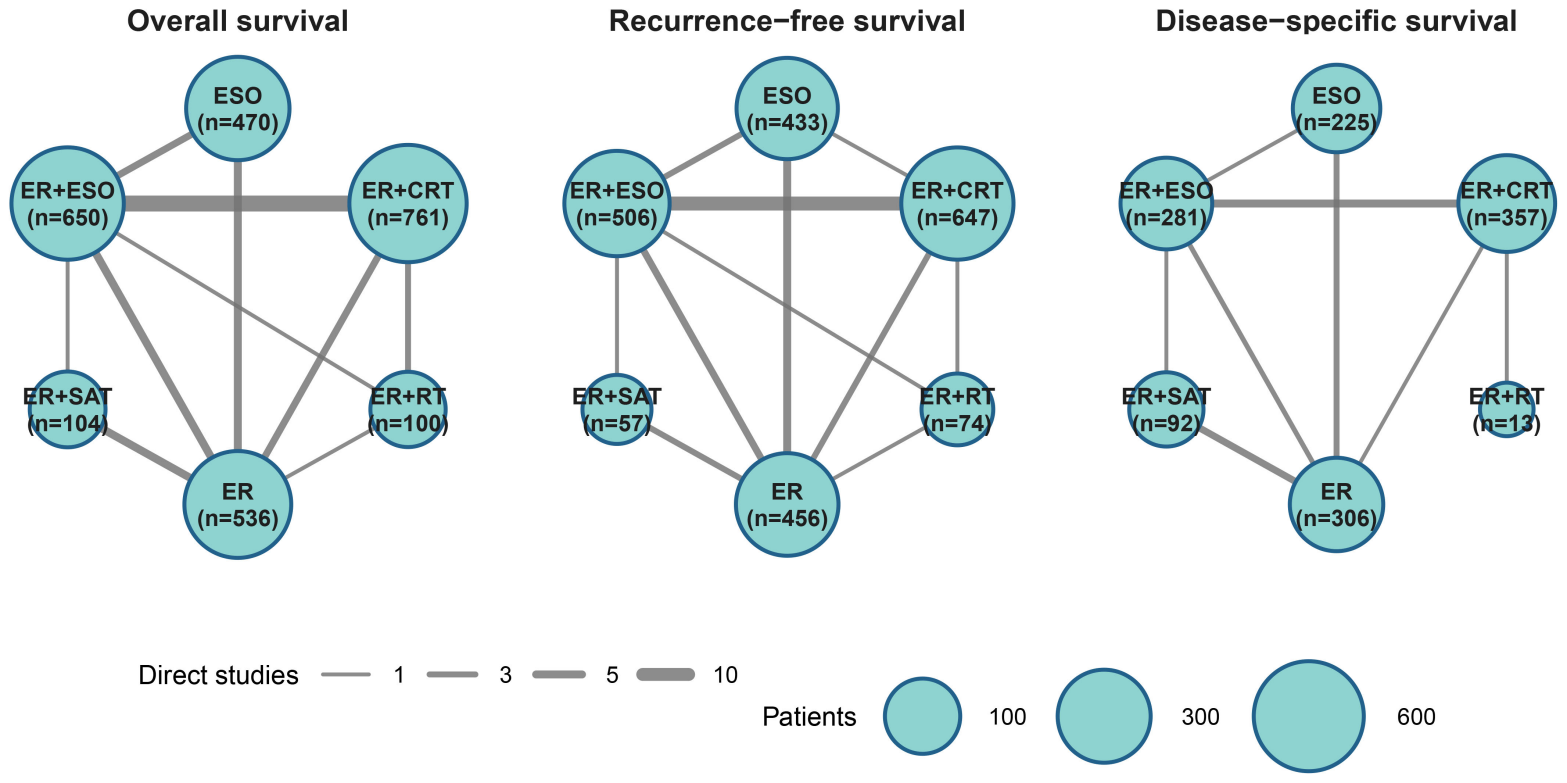
Evidence networks for the survival outcomes. ER, endoscopic resection; ER+ESO, ER followed by esophagectomy; ER+CRT, ER followed by chemoradiotherapy; ER+RT, ER followed by radiotherapy; ER+SAT, ER followed by selective adjuvant therapy; ESO, esophagectomy.

Across the included studies, 2,634 participants contributed to at least one outcome network. The treatment nodes contained 470 participants receiving ESO, 650 receiving ER+ESO, 774 receiving ER+CRT, 536 receiving ER alone, 104 receiving ER+SAT, and 100 receiving ER+RT (**Figure 2**). Age and pathological risk profiles differed across treatment nodes (**Table 1**, **Supplementary Tables 4**, **5**). ER+CRT, ER+RT, and ER+SAT cohorts generally included older patients, while CCI and performance status were incompletely reported. The median study-arm T1b proportion ranged from 71.3% in ER+CRT to 100% in ESO and ER+RT. LVI, positive vertical margin, and poor differentiation were also unevenly reported and distributed. These imbalances were considered when evaluating transitivity and interpreting indirect comparisons.

**Table 1:** Population and tumor characteristics of the included treatment arms.

Study	Treatment	Network N	Age, years	Male, n/N (%)	Comorbidity/performance status	Pathological T category, n/N (%)	Histology, n/N (%)	Tumor size, cm	Tumor location, n (%)	LVI, n/N (%)
An et al., 2025 (25)	ER	95	Mean ± SD: 66.6 ± 7.4	73/95 (76.8%)	Chronic disease: 41/95 (43.2%)	pT1a: 59/95 (62.1%); pT1b: 36/95 (37.9%)	ESCC: 95/95 (100.0%)	Median [IQR]: 3.4 [2.4–4.7]	Ut: 13 (13.7%); Mt: 57 (60.0%); Lt: 25 (26.3%)	LVI/LNM composite
An et al., 2025 (25)	ESO	86	Mean ± SD: 63.4 ± 6.4	68/86 (79.1%)	Chronic disease: 33/86 (38.4%)	pT1a: 25/86 (29.1%); pT1b: 61/86 (70.9%)	ESCC: 86/86 (100.0%)	Median [IQR]: 2 [1.5–3]	Ut: 7 (8.1%); Mt: 50 (58.1%); Lt: 29 (33.7%)	LVI/LNM composite
Beaufort et al., 2024 (26)	ER	17	NR	NR	NR	NR	ESCC: 17/17 (100.0%)	NR	NR	NR
Beaufort et al., 2024 (26)	ER+SAT	15	NR	NR	NR	NR	ESCC: 15/15 (100.0%)	NR	NR	NR
Dermine et al., 2020 (27)	ER+CRT	28	Median: 65	25/28 (89.3%)	NR	pT1a: 1/28 (3.6%); pT1b: 27/28 (96.4%)	ESCC: 26/28 (92.9%); EAC: 2/28 (7.1%)	NR	Ut: 5 (17.9%); Mt: 17 (60.7%); Lt: 6 (21.4%)	8/28 (28.6%)
Dermine et al., 2020 (27)	ER	13	Median: 61	11/13 (84.6%)	NR	pT1a: 8/13 (61.5%); pT1b: 5/13 (38.5%)	ESCC: 10/13 (76.9%); EAC: 3/13 (23.1%)	NR	Ut: 0 (0.0%); Mt: 6 (46.2%); Lt: 7 (53.8%)	3/13 (23.1%)
Emi et al., 2022 (28)	ER+ESO	30	Median: 62	25/30 (83.3%)	NR	pT1a: 6/30 (20.0%); pT1b: 24/30 (80.0%)	ESCC: 30/30 (100.0%)	NR	Ce: 1 (3.3%); Ut: 4 (13.3%); Mt: 16 (53.3%); Lt: 9 (30.0%); Ae: 0 (0.0%)	Ly: 11/30 (36.7%); V: 5/30 (16.7%)
Emi et al., 2022 (28)	ER+CRT	37	Median: 66	34/37 (91.9%)	NR	pT1a: 12/37 (32.4%); pT1b: 25/37 (67.6%)	ESCC: 37/37 (100.0%)	NR	Ce: 4 (10.8%); Ut: 4 (10.8%); Mt: 20 (54.1%); Lt: 8 (21.6%); Ae: 1 (2.7%)	Ly: 15/37 (40.5%); V: 7/37 (18.9%)
Ikeda et al., 2015 (29)	ER+ESO	15	Median [range]: 68 [47–72]	NR	Median CCI: 2	pT1a: 0/15 (0.0%); pT1b: 15/15 (100.0%)	ESCC: 15/15 (100.0%)	Median: 3.9	NR	6/15 (40.0%)
Ikeda et al., 2015 (29)	ER+CRT	11	Median [range]: 69 [49–79]	NR	Median CCI: 4	pT1a: 0/11 (0.0%); pT1b: 11/11 (100.0%)	ESCC: 11/11 (100.0%)	Median: 4.4	NR	8/11 (72.7%)
Ikeda et al., 2015 (29)	ER	17	Median [range]: 77 [65–88]	NR	Median CCI: 5	pT1a: 0/17 (0.0%); pT1b: 17/17 (100.0%)	ESCC: 17/17 (100.0%)	Median: 3.5	NR	3/17 (17.6%)
Kadota et al., 2022 (30)	ER	21	Median [range]: 75 [63–85]	14/21 (66.7%)	NR	pT1a: 2/21 (9.5%); pT1b: 19/21 (90.5%)	ESCC: 21/21 (100.0%)	Median [range]: 2.7 [0.8–7.2]	Ce: 0 (0.0%); Ut: 0 (0.0%); Mt: 10 (47.6%); Lt: 11 (52.4%)	7/21 (33.3%)
Kadota et al., 2022 (30)	ER+ESO	18	Median [range]: 68 [57–79]	16/18 (88.9%)	NR	pT1a: 2/18 (11.1%); pT1b: 16/18 (88.9%)	ESCC: 18/18 (100.0%)	Median [range]: 2.95 [1.2–7.7]	Ce: 1 (5.6%); Ut: 1 (5.6%); Mt: 12 (66.7%); Lt: 4 (22.2%)	12/18 (66.7%)
Kadota et al., 2022 (30)	ER+CRT	50	Median [range]: 68.5 [46–82]	39/50 (78.0%)	NR	pT1a: 10/50 (20.0%); pT1b: 40/50 (80.0%)	ESCC: 50/50 (100.0%)	Median [range]: 3.2 [0.6–7.3]	Ce: 0 (0.0%); Ut: 11 (22.0%); Mt: 30 (60.0%); Lt: 9 (18.0%)	29/50 (58.0%)
Kanie et al., 2021 (31)	ER+CRT	52	Median [range]: 65 [50–77]	46/52 (88.5%)	Median CCI [range]: 0 [0–6]	pT1a: 19/52 (36.5%); pT1b: 33/52 (63.5%)	ESCC: 52/52 (100.0%)	Median [range]: 1.5 [0.4–6.3]	Ut: 9 (17.3%); Mt: 30 (57.7%); Lt: 13 (25.0%)	Ly: 23/52 (44.2%); V: 9/52 (17.3%)
Kanie et al., 2021 (31)	ER+ESO	56	Median [range]: 61.5 [46–79]	45/56 (80.4%)	Median CCI [range]: 1 [0–4]	pT1a: 18/56 (32.1%); pT1b: 36/56 (64.3%); pT2+: 2/56 (3.6%)	ESCC: 56/56 (100.0%)	Median [range]: 2.25 [0.5–7.4]	Ut: 15 (26.8%); Mt: 21 (37.5%); Lt: 20 (35.7%)	Ly: 38/56 (67.9%); V: 11/56 (19.6%)
Katada et al., 2023 (12)	ER+CRT	131	NR	NR	NR	pT1a: 60/131 (45.8%); pT1b: 71/131 (54.2%)	ESCC: 131/131 (100.0%)	NR	NR	NR
Katada et al., 2023 (12)	ER+ESO	58	NR	NR	NR	pT1a: 29/58 (50.0%); pT1b: 29/58 (50.0%)	ESCC: 58/58 (100.0%)	NR	NR	NR
Kim et al., 2023 (32)	ESO	34	Mean ± SD: 62.8 ± 7.3	34/34 (100.0%)	CCI <2: 31/34 (91.2%); ≥2: 3/34 (8.8%)	pT1a: 12/34 (35.3%); pT1b: 22/34 (64.7%)	ESCC: 34/34 (100.0%)	Mean ± SD: 1.9 ± 0.9	Ce/Ut: 1 (2.9%); Mt/Lt/EGJ: 33 (97.1%)	6/34 (17.6%)
Kim et al., 2023 (32)	ER+ESO	34	Mean ± SD: 62.7 ± 6.3	33/34 (97.1%)	CCI <2: 30/34 (88.2%); ≥2: 4/34 (11.8%)	pT1a: 11/34 (32.4%); pT1b: 23/34 (67.6%)	ESCC: 34/34 (100.0%)	Mean ± SD: 2 ± 1.1	Ce/Ut: 0 (0.0%); Mt/Lt/EGJ: 34 (100.0%)	6/34 (17.6%)
Koterazawa et al., 2018 (33)	ER+ESO	28	Median [range]: 66 [47–77]	24/28 (85.7%)	ASA I/II/III: 15/11/2	pT1a: 8/28 (28.6%); pT1b: 20/28 (71.4%)	ESCC: 28/28 (100.0%)	Median [range]: 3.4 [0.9–10]	Ce: 0 (0.0%); Ut: 3 (10.7%); Mt: 20 (71.4%); Lt: 4 (14.3%); Ae: 1 (3.6%)	Ly: 16/28 (57.1%); V: 1/28 (3.6%)
Koterazawa et al., 2018 (33)	ER+CRT	31	Median [range]: 68 [50–81]	24/31 (77.4%)	ASA I/II/III: 16/8/7	pT1a: 3/31 (9.7%); pT1b: 28/31 (90.3%)	ESCC: 31/31 (100.0%)	Median [range]: 2.9 [0.7–8]	Ce: 2 (6.5%); Ut: 4 (12.9%); Mt: 20 (64.5%); Lt: 5 (16.1%); Ae: 0 (0.0%)	Ly: 11/31 (35.5%); V: 3/31 (9.7%)
Kubo et al., 2025 (34)	ER+CRT	29	Median [range]: 67 [52–78]	25/29 (86.2%)	CCI <2: 21/29 (72.4%); ≥2: 8/29 (27.6%)	pT1a: 14/29 (48.3%); pT1b: 15/29 (51.7%)	ESCC: 29/29 (100.0%)	>3 cm: 13/29 (44.8%)	Ce: 0 (0.0%); Ut: 2 (6.9%); Mt: 18 (62.1%); Lt: 7 (24.1%); Ae: 2 (6.9%)	Ly: 19/29 (65.5%); V: 8/29 (27.6%)
Kubo et al., 2025 (34)	ER+ESO	29	Median [range]: 62 [48–82]	24/29 (82.8%)	CCI <2: 20/29 (69.0%); ≥2: 9/29 (31.0%)	pT1a: 7/29 (24.1%); pT1b: 22/29 (75.9%)	ESCC: 29/29 (100.0%)	>3 cm: 15/29 (51.7%)	Ce: 0 (0.0%); Ut: 3 (10.3%); Mt: 18 (62.1%); Lt: 4 (13.8%); Ae: 4 (13.8%)	Ly: 13/29 (44.8%); V: 8/29 (27.6%)
Kudou et al., 2017 (35)	ER+ESO	16	Mean ± SD: 63.1 ± 7.8	16/16 (100.0%)	NR	pT1a: 9/16 (56.2%); pT1b: 7/16 (43.8%)	ESCC: 12/16 (75.0%); EAC: 4/16 (25.0%)	NR	NR	Ly: 10/16 (62.5%); V: 4/16 (25.0%)
Kudou et al., 2017 (35)	ER+SAT	21	Mean ± SD: 71.5 ± 7.1	19/21 (90.5%)	NR	pT1a: 9/21 (42.9%); pT1b: 12/21 (57.1%)	ESCC: 21/21 (100.0%)	NR	NR	Ly: 8/21 (38.1%); V: 4/21 (19.0%)
Lee et al., 2020 (36)	ER	8	NR	NR	NR	pT1a: 0/8 (0.0%); pT1b: 8/8 (100.0%)	ESCC: 8/8 (100.0%)	NR	NR	NR
Lee et al., 2020 (36)	ESO	46	NR	NR	NR	pT1a: 0/46 (0.0%); pT1b: 46/46 (100.0%)	ESCC: 46/46 (100.0%)	NR	NR	NR
Lee et al., 2018 (37)	ER	10	Mean ± SD: 68.6 ± 8	10/10 (100.0%)	ECOG 1/2: 8/2; severe comorbidity: 3/10 (30.0%)	pT1a: 2/10 (20.0%); pT1b: 8/10 (80.0%)	ESCC: 9/10 (90.0%); EAC: 1/10 (10.0%)	Mean ± SD: 1.47 ± 0.74	Ut: 1 (10.0%); Mt: 6 (60.0%); Lt: 3 (30.0%)	2/10 (20.0%)
Lee et al., 2018 (37)	ER+SAT	14	Mean ± SD: 64.7 ± 8.4	12/14 (85.7%)	ECOG 1/2: 12/2; severe comorbidity: 1/14 (7.1%)	pT1a: 1/14 (7.1%); pT1b: 13/14 (92.9%)	ESCC: 14/14 (100.0%)	Mean ± SD: 1.72 ± 0.78	Ut: 1 (7.1%); Mt: 8 (57.1%); Lt: 5 (35.7%)	2/14 (14.3%)
Li et al., 2023 (38)	ER	22	NR	NR	NR	pT1a: 4/22 (18.2%); pT1b: 18/22 (81.8%)	ESCC: 22/22 (100.0%)	NR	NR	NR
Li et al., 2023 (38)	ER+ESO	23	NR	NR	NR	NR	ESCC: 23/23 (100.0%)	NR	NR	NR
Li et al., 20)23 (38)	ER+CRT	7	NR	NR	NR	NR	ESCC: 7/7 (100.0%)	NR	NR	NR
Lin et al., 2025 (39)	ER+RT	13	<72 years: 3/13 (23.1%)	8/13 (61.5%)	NR	pT1a: 0/13 (0.0%); pT1b: 13/13 (100.0%)	EAC: 10/13 (76.9%); ESCC: 3/13 (23.1%)	<5 cm: 11/13 (84.6%); unknown: 2/13 (15.4%)	Ut: 1 (7.7%); Mt: 2 (15.4%); Lt: 9 (69.2%); unknown: 1 (7.7%)	NR
Lin et al., 2025 (39)	ER+CRT	13	<72 years: 6/13 (46.2%)	7/13 (53.8%)	NR	pT1a: 0/13 (0.0%); pT1b: 13/13 (100.0%)	EAC: 9/13 (69.2%); ESCC: 4/13 (30.8%)	<5 cm: 10/13 (76.9%); unknown: 3/13 (23.1%)	Ut: 3 (23.1%); Mt: 2 (15.4%); Lt: 7 (53.8%); unknown: 1 (7.7%)	NR
Liu et al., 2023 (40)	ER	19	Mean ± SD: 61.11 ± 9.15	10/19 (52.6%)	NR	pT1a: 0/19 (0.0%); pT1b: 19/19 (100.0%)	NR	Mean ± SD: 2.15 ± 1.1	Ut: 0 (0.0%); Mt: 14 (73.7%); Lt: 5 (26.3%)	V: 2/19 (10.5%)
Liu et al., 2023 (40)	ESO	19	Mean ± SD: 58.95 ± 9.19	11/19 (57.9%)	NR	pT1a: 0/19 (0.0%); pT1b: 19/19 (100.0%)	NR	Mean ± SD: 2.33 ± 1.91	Ut: 0 (0.0%); Mt: 13 (68.4%); Lt: 6 (31.6%)	V: 3/19 (15.8%)
Minamide et al., 2023 (10)	ER	11	Median [IQR]: 76.0 [65.0–80.0]	10/11 (90.9%)	ECOG 0/1: 6/5; CCI 0–1/≥2: 5/6	pT1a: 6/11 (54.5%); pT1b: 5/11 (45.5%)	ESCC: 11/11 (100.0%)	Median [IQR]: 6.5 [5.9–7.2]	Ut: 0 (0.0%); Mt: 10 (90.9%); Lt: 1 (9.1%); Ae: 0 (0.0%)	Ly: 4/11 (36.4%); V: 3/11 (27.3%)
Minamide et al., 2023 (10)	ER+SAT	10	Median [IQR]: 73.0 [69.0–73.8]	9/10 (90.0%)	ECOG 0/1: 9/1; CCI 0–1/≥2: 8/2	pT1a: 0/10 (0.0%); pT1b: 10/10 (100.0%)	ESCC: 10/10 (100.0%)	Median [IQR]: 5.8 [4.9–6.5]	Ut: 0 (0.0%); Mt: 6 (60.0%); Lt: 4 (40.0%); Ae: 0 (0.0%)	Ly: 3/10 (30.0%); V: 3/10 (30.0%)
Miyata et al., 2021 (41)	ER+ESO	37	Mean ± SD: 59.9 ± 11.7	31/37 (83.8%)	NR	pT1a: 11/37 (29.7%); pT1b: 26/37 (70.3%)	ESCC: 33/37 (89.2%); EAC: 4/37 (10.8%)	NR	Ut: 4 (10.8%); Mt: 20 (54.1%); Lt: 13 (35.1%)	22/37 (59.5%)
Miyata et al., 2021 (41)	ER+CRT	123	Mean ± SD: 66.9 ± 8.9	113/123 (91.9%)	NR	pT1a: 37/123 (30.1%); pT1b: 86/123 (69.9%)	ESCC: 123/123 (100.0%)	NR	Ut: 19 (15.4%); Mt: 69 (56.1%); Lt: 35 (28.5%)	66/123 (53.7%)
Naito et al., 2022 (13)	ER+CRT	33	NR	NR	NR	pT1a: 9/33 (27.3%); pT1b: 24/33 (72.7%)	ESCC: 33/33 (100.0%)	NR	NR	NR
Naito et al., 2022 (13)	ER+ESO	29	NR	NR	NR	pT1a: 11/29 (37.9%); pT1b: 18/29 (62.1%)	ESCC: 29/29 (100.0%)	NR	NR	NR
Nakajo et al., 2019 (42)	ER+SAT	32	Mean ± SD: 77.3 ± 2.2	26/32 (81.2%)	ASA 1–2/3–4: 32/0; CCI 0–1/≥2: 24/8	pT1a: 9/32 (28.1%); pT1b: 23/32 (71.9%)	ESCC: 32/32 (100.0%)	Mean ± SD: 3.96 ± 1.68	Ce/Ut: 6 (18.8%); Mt: 17 (53.1%); Lt/Ae: 9 (28.1%)	Ly: 15/32 (46.9%); V: 11/32 (34.4%)
Nakajo et al., 2019 (42)	ER	41	Mean ± SD: 79.6 ± 3.9	34/41 (82.9%)	ASA 1–2/3–4: 35/6; CCI 0–1/≥2: 17/24	pT1a: 12/41 (29.3%); pT1b: 29/41 (70.7%)	ESCC: 41/41 (100.0%)	Mean ± SD: 4.09 ± 1.70	Ce/Ut: 7 (17.1%); Mt: 24 (58.5%); Lt/Ae: 10 (24.4%)	Ly: 11/41 (26.8%); V: 14/41 (34.1%)
Nishibuchi et al., 2020 (43)	ER+CRT	24	NR	NR	NR	NR	ESCC: 24/24 (100.0%)	NR	NR	NR
Nishibuchi et al., 2020 (43)	ER+RT	13	NR	NR	NR	NR	ESCC: 13/13 (100.0%)	NR	NR	NR
Plum et al., 2018 (44)	ER+ESO	37	NR	NR	NR	pT1a: 0/37 (0.0%); pT1b: 37/37 (100.0%)	NR	NR	NR	NR
Plum et al., 2018 (44)	ESO	37	NR	NR	NR	pT1a: 0/37 (0.0%); pT1b: 37/37 (100.0%)	NR	NR	NR	NR
Song et al., 2021 (45)	ER	23	Median [range]: 68 [52–86]	23/23 (100.0%)	Median CCI [range]: 2 [1–6]	pT1a: 6/23 (26.1%); pT1b: 17/23 (73.9%)	ESCC: 23/23 (100.0%)	Median [range]: 1.5 [0.6–4.3]	Ce: 0 (0.0%); Ut: 1 (4.3%); Mt: 9 (39.1%); Lt: 13 (56.5%); Ae: 0 (0.0%)	8/23 (34.8%)
Song et al., 2021 (45)	ER+ESO	29	Median [range]: 62 [52–73]	28/29 (96.6%)	Median CCI [range]: 2 [1–4]	pT1a: 1/29 (3.4%); pT1b: 28/29 (96.6%)	ESCC: 29/29 (100.0%)	Median [range]: 1.8 [0.7–3.1]	Ce: 0 (0.0%); Ut: 2 (6.9%); Mt: 12 (41.4%); Lt: 15 (51.7%); Ae: 0 (0.0%)	12/29 (41.4%)
Suzuki et al., 2022 (46)	ER+CRT	34	Median [range]: 69 [50–80]	29/34 (85.3%)	NR	pT1a: 11/34 (32.4%); pT1b: 23/34 (67.6%)	ESCC: 34/34 (100.0%)	NR	Ce/Ut: 9 (26.5%); Mt: 15 (44.1%); Lt: 10 (29.4%); Ae: 0 (0.0%)	Ly: 9/34 (26.5%); V: 12/34 (35.3%)
Suzuki et al., 2022 (46)	ER+ESO	26	Median [range]: 65 [45–78]	24/26 (92.3%)	NR	pT1a: 8/26 (30.8%); pT1b: 18/26 (69.2%)	ESCC: 26/26 (100.0%)	NR	Ce/Ut: 6 (23.1%); Mt: 15 (57.7%); Lt: 4 (15.4%); Ae: 1 (3.8%)	Ly: 13/26 (50.0%); V: 8/26 (30.8%)
Takahashi et al., 2018 (47)	ER+SAT	12	Mean ± SD: 66.8 ± 8.2	12/12 (100.0%)	NR	pT1a: 7/12 (58.3%); pT1b: 5/12 (41.7%)	ESCC: 12/12 (100.0%)	Mean ± SD: 3.15 ± 1.21	NR	LVI/INFc composite
Takahashi et al., 2018 (47)	ER	11	Mean ± SD: 71.1 ± 7.3	10/11 (90.9%)	NR	pT1a: 7/11 (63.6%); pT1b: 4/11 (36.4%)	ESCC: 11/11 (100.0%)	Mean ± SD: 3.75 ± 2.04	NR	LVI/INFc composite
Takeuchi et al., 2018 (48)	ESO	51	NR	NR	NR	NR	NR	NR	NR	NR
Takeuchi et al., 2018 (48)	ER+ESO	19	Median [range]: 62 [45–81]	17/19 (89.5%)	Performance status 0/1: 14/5	pT1a: 2/19 (10.5%); pT1b: 16/19 (84.2%); pT2+: 1/19 (5.3%)	NR	NR	Ce: 0 (0.0%); Ut: 0 (0.0%); Mt: 12 (63.2%); Lt: 7 (36.8%)	NR
Takeuchi et al., 2018 (48)	ER+CRT	13	NR	NR	NR	NR	NR	NR	NR	NR
Tanaka et al., 2024 (49)	ER+ESO	57	Median [IQR]: 65 [60–69]	46/57 (80.7%)	Median CCI [IQR]: 0 [0–1]	pT1a: 11/57 (19.3%); pT1b: 46/57 (80.7%)	ESCC: 54/57 (94.7%); other: 3/57 (5.3%)	Median [IQR]: 3 [1.7–4.8]	Ut: 7 (12.3%); Mt: 30 (52.6%); Lt: 20 (35.1%)	Ly: 36/57 (63.2%); V: 22/57 (38.6%)
Tanaka et al., 2024 (50)	ER+CRT	125	Median [IQR]: 68 [62–73]	116/125 (92.8%)	Median CCI [IQR]: 0 [0–2]	pT1a: 41/125 (32.8%); pT1b: 84/125 (67.2%)	ESCC: 120/125 (96.0%); other: 5/125 (4.0%)	Median [IQR]: 2.1 [1.5–3.5]	Ut: 23 (18.4%); Mt: 67 (53.6%); Lt: 35 (28.0%)	Ly: 59/125 (47.2%); V: 41/125 (32.8%)
Tanaka et al., 2024 (49)	ER+RT	38	Median [IQR]: 73 [68–78]	36/38 (94.7%)	Median CCI [IQR]: 1 [0–3]	pT1a: 14/38 (36.8%); pT1b: 24/38 (63.2%)	ESCC: 36/38 (94.7%); other: 2/38 (5.3%)	Median [IQR]: 2.9 [1.8–4.5]	Ut: 3 (7.9%); Mt: 26 (68.4%); Lt: 9 (23.7%)	Ly: 19/38 (50.0%); V: 27/38 (71.1%)
Tanaka et al., 2019 (50)	ER+ESO	19	Median [range]: 63 [43–76]	15/19 (78.9%)	Median CCI [range]: 0 [0–5]	pT1a: 0/19 (0.0%); pT1b: 19/19 (100.0%)	ESCC: 19/19 (100.0%)	Median [range]: 3.8 [0.7–8.8]	Ce: 0 (0.0%); Ut: 3 (15.8%); Mt: 11 (57.9%); Lt: 4 (21.1%); Ae: 0 (0.0%); EGJ: 1 (5.3%)	17/19 (89.5%)
Tanaka et al., 2019 (50)	ER+CRT	33	Median [range]: 65 [50–79]	32/33 (97.0%)	Median CCI [range]: 0 [0–4]	pT1a: 0/33 (0.0%); pT1b: 33/33 (100.0%)	ESCC: 33/33 (100.0%)	Median [range]: 2.2 [0.5–8]	Ce: 1 (3.0%); Ut: 3 (9.1%); Mt: 21 (63.6%); Lt: 8 (24.2%); Ae: 0 (0.0%); EGJ: 0 (0.0%)	16/33 (48.5%)
Wang et al., 2022 (51)	ER	21	NR	NR	NR	pT1a: 0/21 (0.0%); pT1b: 21/21 (100.0%)	ESCC: 21/21 (100.0%)	NR	NR	NR
Wang et al., 2022 (51)	ESO	28	NR	NR	NR	pT1a: 0/28 (0.0%); pT1b: 28/28 (100.0%)	ESCC: 28/28 (100.0%)	NR	NR	NR
Wang et al., 2018 (52)	ER+ESO	48	Mean ± SD: 60.4 ± 7.8	31/48 (64.6%)	NR	pT1a: 24/48 (50.0%); pT1b: 24/48 (50.0%)	ESCC: 43/48 (89.6%); EAC: 5/48 (10.4%)	Mean ± SD: 3.2 ± 2	Ce: 0 (0.0%); Ut: 5 (10.4%); Mt: 34 (70.8%); Lt: 9 (18.8%); Ae: 0 (0.0%)	0/48 (0.0%)
Wang et al., 2018 (52)	ESO	96	Mean ± SD: 60.4 ± 7.3	67/96 (69.8%)	NR	pT1a: 41/96 (42.7%); pT1b: 55/96 (57.3%)	ESCC: 87/96 (90.6%); EAC: 9/96 (9.4%)	Mean ± SD: 2.9 ± 1.5	Ce: 0 (0.0%); Ut: 12 (12.5%); Mt: 64 (66.7%); Lt: 20 (20.8%); Ae: 0 (0.0%)	1/96 (1.0%)
Xue et al., 2023 (53)	ER+ESO	42	Median [IQR]: 60.5 [55–64]	37/42 (88.1%)	NR	pT1a: 0/42 (0.0%); pT1b: 42/42 (100.0%)	ESCC: 42/42 (100.0%)	NR	Ut: 7 (16.7%); Mt: 9 (21.4%); Lt: 26 (61.9%)	13/42 (31.0%)
Xue et al., 2023 (54)	ER	124	Median [IQR]: 62 [57–67]	93/124 (75.0%)	NR	pT1a: 0/124 (0.0%); pT1b: 124/124 (100.0%)	ESCC: 124/124 (100.0%)	NR	Ut: 17 (13.7%); Mt: 42 (33.9%); Lt: 65 (52.4%)	16/124 (12.9%)
Yang et al., 2023 (6)	ER+RT	36	<65 years: 22/36 (61.1%)	29/36 (80.6%)	ECOG 0/1: 25/11	pT1a: 0/36 (0.0%); pT1b: 36/36 (100.0%)	ESCC: 36/36 (100.0%)	≥3 cm: 23/36 (63.9%)	Ce: 0 (0.0%); Ut: 6 (16.7%); Mt: 17 (47.2%); Lt: 13 (36.1%); EGJ: 0 (0.0%)	12/36 (33.3%)
Yang et al., 2023 (6)	ER	36	<65 years: 28/36 (77.8%)	28/36 (77.8%)	ECOG 0/1: 25/11	pT1a: 0/36 (0.0%); pT1b: 36/36 (100.0%)	ESCC: 36/36 (100.0%)	≥3 cm: 22/36 (61.1%)	Ce: 0 (0.0%); Ut: 5 (13.9%); Mt: 19 (52.8%); Lt: 12 (33.3%); EGJ: 0 (0.0%)	9/36 (25.0%)
Zhang et al., 2024 ( 55)	ER	47	Mean ± SD: 66.17 ± 6.82	32/47 (68.1%)	CCI 0/1/≥2: 41/6/0	pT1a: 0/47 (0.0%); pT1b: 47/47 (100.0%)	ESCC: 47/47 (100.0%)	Mean ± SD: 1.95 ± 1.29	Ut: 12 (25.5%); Mt: 20 (42.6%); Lt: 15 (31.9%)	4/47 (8.5%)
Zhang et al., 2024 (55)	ESO	73	Mean ± SD: 64.38 ± 7.64	54/73 (74.0%)	CCI 0/1/≥2: 67/5/1	pT1a: 0/73 (0.0%); pT1b: 73/73 (100.0%)	ESCC: 73/73 (100.0%)	Mean ± SD: 2.06 ± 1.04	Ut: 15 (20.5%); Mt: 40 (54.8%); Lt: 18 (24.7%)	5/73 (6.8%)

*Note.* Ae, abdominal esophagus; ASA, American Society of Anesthesiologists physical status; CCI, Charlson Comorbidity Index; Ce, cervical esophagus; EAC, esophageal adenocarcinoma; ECOG, Eastern Cooperative Oncology Group; EGJ, esophagogastric junction; ER, endoscopic resection; ESCC, esophageal squamous cell carcinoma; INFc, infiltrative growth pattern c; IQR, interquartile range; Lt, lower thoracic esophagus; LVI, lymphovascular invasion; Ly, lymphatic invasion; Mt, middle thoracic esophagus; NR, not reported; SD, standard deviation; Ut, upper thoracic esophagus; V, venous invasion.

### Overall Survival

**Figure 3:**
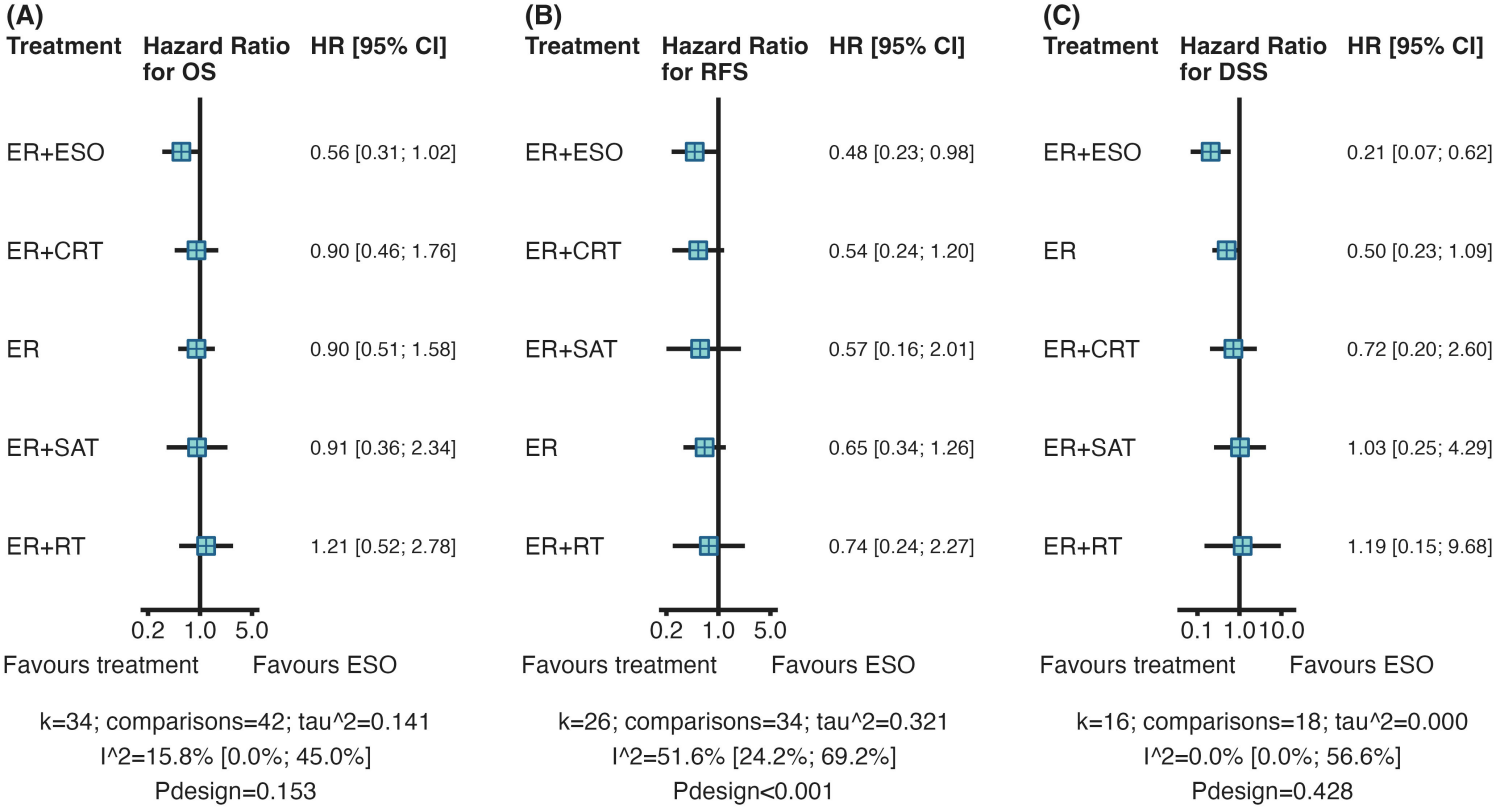
Random-effects network estimates versus esophagectomy for **(A)** overall survival, **(B)** recurrence-free survival, and **(C)** disease-specific survival. Squares show hazard ratios, and horizontal lines show 95% confidence intervals.

The OS network included 34 studies, 2,621 participants, six treatment strategies, and 42 contrasts. In the primary random-effects model, the point estimate favored ER+ESO over ESO, but the 95% confidence interval (CI) included the null value (HR, 0.562; 95% CI, 0.311–1.015; P = 0.056; **Figure 3**). Compared with ESO, the HRs were 0.897 (95% CI, 0.508–1.583) for ER, 0.897 (95% CI, 0.457–1.761) for ER+CRT, 1.208 (95% CI, 0.524–2.784) for ER+RT, and 0.912 (95% CI, 0.356–2.340) for ER+SAT. None of the remaining estimates was statistically significant. Heterogeneity was low (τ²=0.141; I²=15.8%), and the design-by-treatment interaction test did not detect global inconsistency (P = 0.153; **Supplementary Table 7**).

### Recurrence-free survival

The RFS network included 26 studies, 2,173 participants, six treatment strategies, and 34 contrasts. In the primary random-effects model, the point estimate favored ER+ESO over ESO, and the 95% CI narrowly excluded the null value (HR, 0.480; 95% CI, 0.235–0.983; P = 0.045; **Figure 3**). Compared with ESO, the HRs were 0.653 (95% CI, 0.338–1.258) for ER, 0.535 (95% CI, 0.240–1.195) for ER+CRT, 0.743 (95% CI, 0.243–2.271) for ER+RT, and 0.568 (95% CI, 0.160–2.010) for ER+SAT. None of the remaining estimates was statistically significant. Heterogeneity was moderate (τ²=0.321; I²=51.6%), and the design-by-treatment interaction test detected global inconsistency (Q = 30.43; P<0.001). After diagnostic detachment of the ER-versus-ER+ESO design, global inconsistency was no longer statistically significant (Q = 9.01; P = 0.342). This identified an influential evidence structure but did not justify *post hoc* exclusion of the contributing studies (**Supplementary Table 11**).

### Disease-specific survival

**Figure 4:**
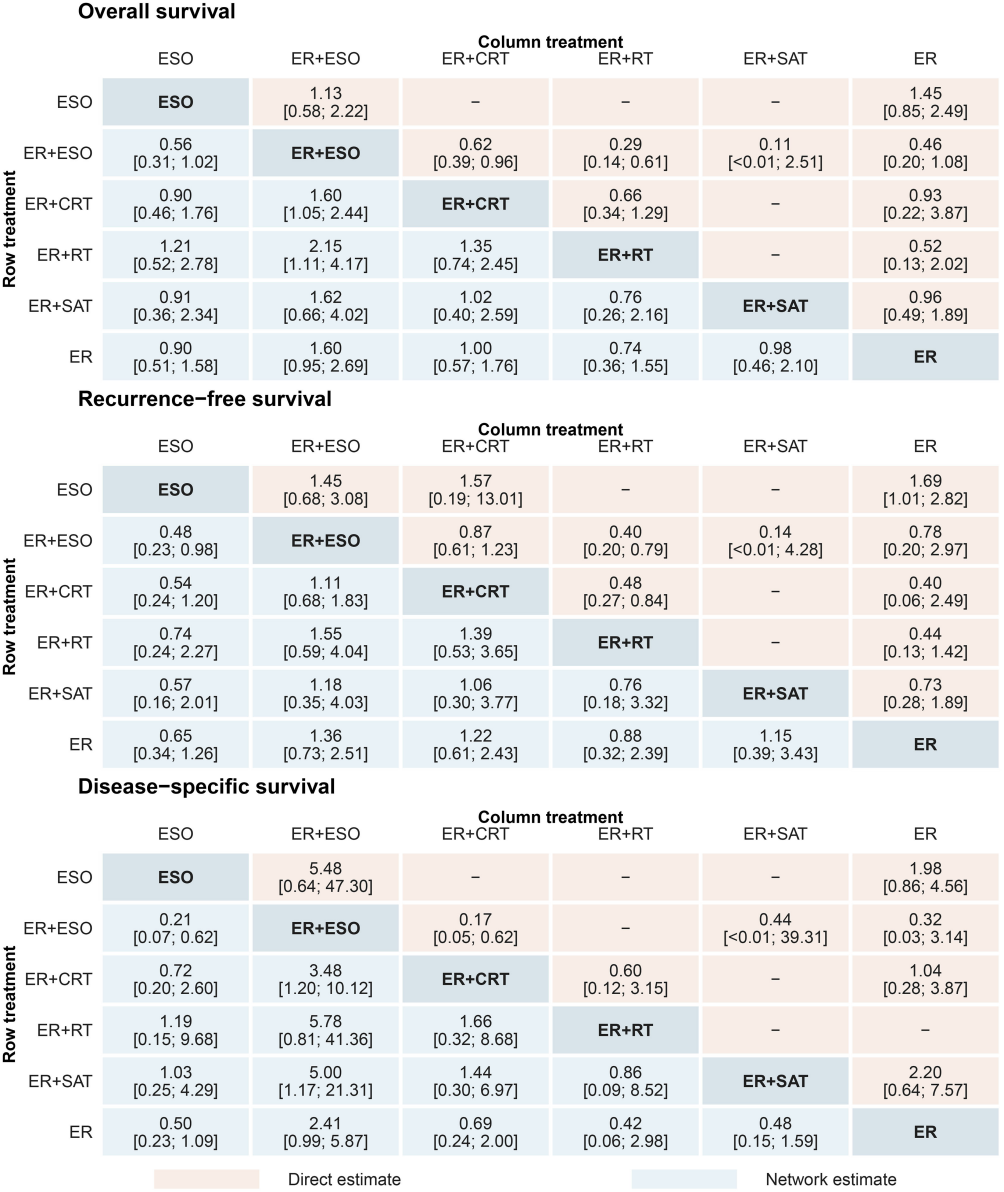
Complete league tables for the survival outcomes. Upper triangles show direct random−effects estimates; lower triangles show network random−effects estimates. Each cell compares the row treatment with the column treatment; HR <1 favours the row treatment.

The DSS network included 16 studies, 1,274 participants, six treatment strategies, and 18 contrasts. In the primary random-effects model, the point estimate favored ER+ESO over ESO, and the 95% CI excluded the null value (HR, 0.206; 95% CI, 0.069–0.619; P = 0.005; **Figure 3**). Compared with ESO, the HRs were 0.497 (95% CI, 0.226–1.093) for ER, 0.718 (95% CI, 0.199–2.596) for ER+CRT, 1.192 (95% CI, 0.147–9.680) for ER+RT, and 1.031 (95% CI, 0.248–4.286) for ER+SAT. None of the remaining estimates was statistically significant. Estimated heterogeneity was negligible (τ²<0.001; I²=0%), and the design-by-treatment interaction test did not detect global inconsistency (P = 0.428; **Supplementary Table 7**). Across all estimable comparisons, node-splitting analyses did not detect statistically significant disagreement between direct and indirect evidence for OS, RFS, or DSS (**Supplementary Table 6**). Complete network estimates are presented in **Figure 4**.

### Adverse events

Eight studies reported grade 3 or higher AEs across 14 treatment arms (25, 27, 30, 33, 36, 48, 51, 55) (**Table 2**). Some reports provided the number of unique patients with at least one event, whereas others provided category-specific event counts that could include more than one event per patient. Grading systems and reporting periods also differed. Formal pooling was therefore not considered valid, and the available data did not support a reliable comparative ranking of treatment safety.

**Table 2:** Severe adverse events reported in the included studies.

Study	Treatment	Grading system	Reported grade ≥3 burden	Cohort N
An et al., 2025 (25)	ER	Clavien–Dindo	36/95 patients (37.9%)	95
An et al., 2025 (25)	ESO	Clavien–Dindo	19/86 patients (22.1%)	86
Dermine et al., 2020 (27)	ER+CRT	CTCAE v5.0	4 patients	28
Kadota et al., 2022 (30)	ER+ESO	Clavien–Dindo v2.0	6 events in 5/18 patients (27.8%)	18
Kadota et al., 2022 (30)	ER+CRT	CTCAE v5.0	29 category-specific events; unique patients NR	50
Koterazawa et al., 2018 (33)	ER+CRT	CTCAE v4.0	4 category-specific events; unique patients NR	31
Lee et al., 2020 (36)	ER	Clavien–Dindo	2 events in 2/63 patients (3.2%)	63
Lee et al., 2020 (36)	ESO	Clavien–Dindo	22 events in 19/93 patients (20.4%)	93
Takeuchi et al., 2018 (48)	ER+ESO	Clavien–Dindo	5 events in 5/19 patients (26.3%)	19
Takeuchi et al., 2018 (48)	ESO	Clavien–Dindo	21 events in 17/54 patients (31.5%)	54
Wang et al., 2022 (51)	ER	Clavien–Dindo	6/40 patients (15.0%)	40
Wang et al., 2022 (51)	ESO	Clavien–Dindo	10/32 patients (31.3%)	32
Zhang et al., 2024 (55)	ER	Clavien–Dindo	8/47 patients (17.0%)	47
Zhang et al., 2024 (55)	ESO	Clavien–Dindo	15/73 patients (20.5%)	73

Category-specific counts may include more than one event per patient. CTCAE, Common Terminology Criteria for Adverse Events; ER, endoscopic resection; ESO, esophagectomy; NR, not reported.

### Subgroup analysis and network meta-regression

The study-level pathological-stage subgroup analysis did not show statistically significant differences in treatment effects between pT1b-only and mixed pT1a/pT1b studies for any estimable comparison with ESO (P for interaction=0.349–0.902 for OS and 0.352–0.575 for RFS; **Supplementary Table 8**, **Supplementary Figure 3**). For ER+ESO versus ESO, the OS HR was 0.362 (95% CI, 0.113–1.153) in mixed-stage studies and 0.713 (95% CI, 0.314–1.618) in pT1b-only studies (P for interaction=0.349). The corresponding RFS HRs were 0.277 (95% CI, 0.110–0.698) and 0.486 (95% CI, 0.086–2.745), respectively (P for interaction=0.575). The pT1b-only DSS network was not estimable.

**Figure 5:**
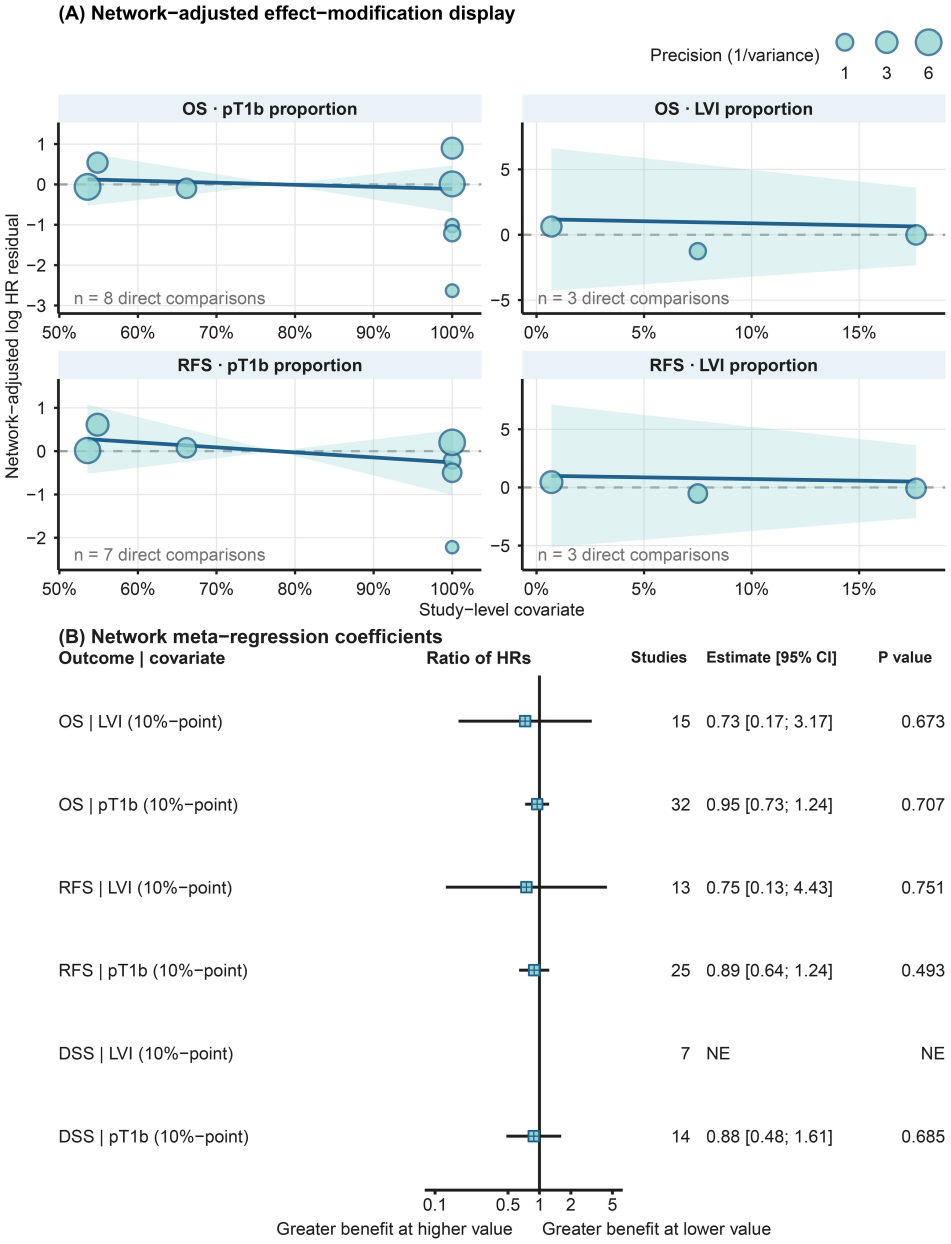
Exploratory network meta−regression of study−level effect modifiers. Panel **(A)** shows network−adjusted effect−modification displays; panel **(B)** summarizes meta−regression coefficients.

Exploratory common-slope network meta-regression did not provide evidence that study-level pathological burden modified relative treatment effects (**Figure 5**, **Supplementary Table 9**). Per 10-percentage-point increase in pT1b proportion, the ratio of HRs was 0.950 for OS (95% CI, 0.730–1.238; P = 0.707), 0.890 for RFS (95% CI, 0.639–1.241; P = 0.493), and 0.882 for DSS (95% CI, 0.482–1.615; P = 0.685). Estimates for LVI proportion remained imprecise, with ratios of HRs of 0.729 for OS (95% CI, 0.168–3.170; P = 0.673) and 0.750 for RFS (95% CI, 0.127–4.429; P = 0.751). The DSS LVI model was not estimated because only seven studies were eligible.

Full network estimates for each sensitivity scenario are presented in **Supplementary Figure 4** and **Supplementary Table 10**. Precision and statistical significance varied by model, outcome, and comparison. For ER+ESO versus ESO, the common-effect estimate was statistically significant for OS but not RFS, whereas the primary random-effects estimate was statistically significant for RFS but not OS. Analyses restricted to verified propensity-score-matched effect cohorts were imprecise, and the DSS network was disconnected. In the post-ER network, ER+ESO was associated with improved OS compared with ER alone (HR, 0.478; 95% CI, 0.262–0.871; P = 0.016), whereas the other comparisons with ER alone were not statistically significant. This post-ER analysis should therefore be interpreted as evaluating whether additional treatment after ER improves outcomes, rather than as a direct comparison between sequential ER+ESO and upfront ESO.

## Discussion

This network meta-analysis of 34 studies and 2,634 patients with pathologically high-risk superficial esophageal cancer compared six multidisciplinary treatment pathways: ER, ESO, ER+CRT, ER+ESO, ER+RT, and ER+SAT. ER+ESO was associated with favorable RFS and DSS estimates compared with ESO, while the OS estimate showed the same direction but included the null. No other strategy differed significantly from ESO. The RFS network showed moderate heterogeneity and significant global inconsistency, whereas inconsistency was not detected for OS or DSS. Clinical and methodological differences among studies, particularly in baseline patient characteristics and pathological definitions, may have contributed to the inconsistency observed in the RFS network. However, these differences reflect the real-world setting in which treatment decisions are made for patients with heterogeneous high-risk pathological features. To our knowledge, this is the first network meta-analysis to compare these sequential treatment pathways across OS, RFS, and DSS. Its main contribution is to provide an outcome-oriented synthesis of the available evidence, helping to clarify which strategies are associated with lower recurrence or disease-specific mortality and where the certainty of comparison remains limited.

ER+ESO represents a clinically distinct two-stage pathway rather than a simple combination of two curative procedures. In current practice, ER is often selected for cT1a lesions with a low estimated risk of recurrence and lymph-node metastasis (11, 58). However, preoperative prediction of invasion depth remains imperfect. Narrow-band imaging achieved reported diagnostic accuracies of 55.2% for cT1a-M3/cT1b-SM1 and 67.5% for cT1b-SM2 or deeper disease (48). In a retrospective analysis of 22,123 patients, Rice et al. found that clinically staged superficial cancers had poorer survival than expected from the corresponding pathological categories (59). Such discrepancies may lead to pathological upstaging after ER and create uncertainty regarding subsequent treatment. ER provides precise information on invasion depth, margins, differentiation, and LVI, but it does not sample regional lymph nodes. When high-risk pathological findings are identified, subsequent ESO can provide definitive resection together with regional lymphadenectomy and pathological nodal staging. This sequence explains why ER+ESO remains an important curative option for medically fit patients (18, 19, 33, 34).

The favorable association observed for ER+ESO though more likely reflects the entire staged treatment pathway rather than a direct improvement in the efficacy of subsequent ESO by ER itself. Previous studies comparing ER+ESO with primary ESO focused mainly on whether preceding ER altered long-term survival or postoperative outcomes (32, 52). These reports generally indicated that preceding ER did not compromise subsequent surgical outcomes, but they do not establish superiority of this sequential pathway. Patients proceeding from ER to surgery have already undergone two stages of clinical selection, first for endoscopic treatment and then for their ability and willingness to undergo ESO. They may have had earlier-detected, smaller, or more localized tumors, better functional status, or treatment at experienced centres. ER may also have reduced visible primary tumor burden and provided pathological information that guided subsequent treatment. Conversely, primary ESO cohorts often contained a greater burden of pT1b disease or lesions considered unsuitable for endoscopic treatment from the outset, while a positive vertical margin cannot be defined before upfront surgery in the same way as after ER. Differences in calendar period may also matter because ER+ESO cohorts were often treated more recently than primary surgical cohorts. Taken together, these differences indicate that this treatment nodes were not fully exchangeable with respect to diagnostic timing, patient fitness, pathological burden, or high-risk features such as LVI or vertical margin status, which is consistent with the baseline and pathological imbalances observed across treatment nodes (**Table 1**, **Supplementary Tables 4**, **5**). These differences may help explain why ER+ESO appeared favorable in survival analyses, although its overall clinical value remains difficult to define fully because comparative data on postoperative morbidity and quality of life were limited.

ER+CRT represents a distinct multidisciplinary pathway that combines endoscopic removal of the primary lesion and pathological risk stratification with CRT, thereby preserving the esophagus. In our network, ER+CRT did not differ significantly from ESO in OS, RFS, or DSS (**Figure 3**). Because the contributing studies were neither designed nor powered to establish non-inferiority, these findings indicate uncertainty in comparative effectiveness rather than therapeutic equivalence. ER+CRT nevertheless remains clinically relevant for patients who wish to avoid morbidity associated with esophagectomy or whose operative risk is high. ER before CRT serves both therapeutic and diagnostic purposes. It removes the visible primary lesion and provides a resection specimen for assessing high-risk pathological features. CRT could subsequently target microscopic residual disease and occult regional nodal disease beyond the reach of ER alone. Definitive CRT without ER was therefore excluded because it does not provide pathological risk stratification from a resection specimen and concerns a clinically staged rather than pathologically selected population.

In JCOG0502, definitive CRT was compared with surgery in patients with cT1bN0M0 disease. Five-year OS was 85.5% after CRT and 86.5% after surgery (adjusted HR, 1.052; 95% CI, 0.67–1.64), whereas five-year progression-free survival (PFS) was lower after CRT (71.6% versus 81.7%; HR, 1.478; 95% CI, 1.010–2.162) (60). The coexistence of similar OS and lower PFS suggests that intensive surveillance and effective salvage treatment may have contributed to the preservation of OS despite less recurrence control. These findings provide relevant clinical context but cannot be directly extrapolated to the present network of treatments after ER. JCOG0508 more closely reflects the pathway evaluated here and supports the feasibility of diagnostic ER followed by selected CRT according to pathological findings. However, its single-arm design precludes direct comparison with ESO (9).

Patient fitness is similarly important when choosing among ER+CRT or ER+RT against ESO. In the included studies, patients receiving ER+CRT or ER+RT were often older or less fit, and poor performance status influenced treatment selection (29). Among elderly patients after non-curative endoscopic submucosal dissection (ESD), a Charlson Comorbidity Index (CCI) of at least 2 was independently associated with mortality, and most deaths were unrelated to esophageal cancer (42). These differences may influence OS more strongly than RFS or DSS. RT alone may be considered when chemotherapy cannot be tolerated, but the available evidence remains limited. Compared with ESO, the estimates for ER+RT were imprecise for OS (HR, 1.208; 95% CI, 0.524–2.784), RFS (HR, 0.743; 95% CI, 0.243–2.271), and DSS (HR, 1.192; 95% CI, 0.147–9.680) (**Figure 3**). These wide intervals reflect small samples and few events rather than equivalence with CRT or surgery.

In a study of elderly patients with esophageal cancer receiving CRT, a CCI of at least 2 was associated with grade 3 or 4 toxicity (61). The survival estimates for ER+CRT and ER+RT therefore should be considered alongside their effects on late toxicity and esophageal function for such patients. Although ER can achieve local control exceeding 90% in selected superficial lesions (27, 62), extensive resection may create a mucosal defect that predisposes to stenosis. Subsequent CRT or RT may further increase inflammation and fibrosis. Previous study showed that esophageal strictures were reported in 3 of 34 patients after ESD followed by CRT (46), chronic stenosis occurred in 2 of 52 patients receiving CRT (31), and grade 2 stenosis was reported in 7 of 37 patients receiving radiotherapy (43). Most patients with grade 2 stenosis had lesions involving at least three-quarters of the esophageal circumference. Clinically relevant stenosis may require repeated endoscopic dilation and impair swallowing and quality of life. Preserving the esophagus therefore does not always preserve normal swallowing function. However, these rates cannot be compared directly because the extent of ER, radiation dose and field, and toxicity definitions differed among studies. Moreover, some studies reported numbers of adverse events rather than numbers of affected patients, preventing a reliable quantitative comparison between treatment pathways.

In addition, ESO removes the esophagus but permits lymph node dissection and pathological staging. ER+CRT and ER+RT preserve the esophagus, but irradiation may also affect both long-term function and subsequent treatment. Late cardiopulmonary toxicity may occur after irradiation (63). Evidence from definitive CRT suggests that prior irradiation can limit reirradiation and make salvage esophagectomy more difficult because of fibrosis (64, 65). These findings may not apply directly to every regimen used after ER because the effects depend on the radiation dose and field. This issue remains relevant because patients with upper ESCC have an increased risk of synchronous or metachronous squamous cell carcinoma of the upper aerodigestive tract, consistent with field cancerization (66). Reirradiation or salvage surgery may still be possible in selected patients, but its feasibility should be considered before the initial treatment is selected. Consequently, the choice between esophagectomy and organ-preserving treatment cannot be reduced to survival estimates or the incidence of grade 3 or higher adverse events. Taken together, ER+CRT represents a pathology guided organ-preserving strategy, whereas ER+RT may be considered for selected patients unable to tolerate chemotherapy, although the supporting evidence remains limited. Their clinical value cannot be judged from our survival estimates or conventionally reported adverse events alone, because swallowing function, late toxicity, and the feasibility of subsequent treatment are also important.

ER followed by surveillance should not be interpreted as a standardized definitive treatment equivalent to surgery, and it neither treats occult nodal disease nor provides pathological lymph node staging. In the present network, ER did not differ significantly from ESO in OS, RFS, or DSS, but the confidence intervals were wide and do not establish equivalent cancer control. The ER node also combined clinically different patients. Some may have a relatively limited residual risk after resection, whereas others received no planned additional treatment because of comorbidity, operative risk, or personal preference. Their subsequent outcomes may therefore reflect both patient selection and the effectiveness of salvage treatment rather than ER alone. For fit patients with high-risk pathology, the central question is whether regional disease should be treated before recurrence develops. For patients who cannot tolerate or decline additional treatment, surveillance represents a deliberate management strategy that requires structured endoscopic and imaging follow-up, predefined criteria for salvage treatment, and consideration of salvage feasibility from the outset.

ER+SAT should likewise be understood as a heterogeneous treatment node rather than a standardized regimen. Depending on the study and pathological findings, this node included ESO, CRT, RT, chemotherapy, lymph node dissection, observation, or combinations of these approaches. Such heterogeneity reflects clinical practice, in which further management after ER is selected according to pathological risk, patient fitness, and preference. However, it also means that the pooled ER+SAT estimate has no single therapeutic interpretation and may dilute the effect of an effective treatment. Excluding ER+SAT did not materially alter the direction of the ER+ESO estimates, suggesting that this heterogeneous node did not drive the principal finding. High-risk pathological findings often prompt consideration of additional treatment after ER, but the ER+SAT estimate cannot determine whether all such patients require further treatment, or which treatment should be preferred.

The heterogeneity within the ER and ER+SAT nodes exemplifies a broader limitation of the entire network. In the included observational studies, treatment selection was inherently related to pathological risk, age, comorbidity, operative fitness, and patient preference. Sensitivity analyses generally preserved the direction of the principal estimates, although precision and statistical significance varied with model choice and network composition. Subgroup analyses and network meta-regression did not identify statistically significant effect modification by pathological stage or study-level T1b and LVI proportions, but the aggregate data provided limited assessment of differences between treatment arms. Overall, this network meta-analysis offers a structured comparison of survival outcomes across currently available treatment pathways based on endoscopic resection and surgery. The broader clinical value of each pathway also depends on pathological risk, operative fitness, treatment burden, quality of life, and the options available if recurrence occurs.

## Limitations

Several limitations should be considered. Most evidence was retrospective, and definitions of high-risk pathology and treatment protocols varied across studies. Reporting of comorbidity, performance status, pathological features, and adverse events was incomplete or inconsistent. Some studies reported numbers of adverse events rather than numbers of affected patients. The RFS network showed global inconsistency, and diagnostic analysis identified the ER versus ER+ESO design as an influential evidence structure. Comparisons for several treatment pathways were also based on small samples and few events. Future prospective studies should use consistent pathological definitions, clearly defined treatment pathways, and standardized reporting of survival, adverse events, quality of life, and functional outcomes.

## Conclusion

ER+ESO was associated with favorable RFS and DSS estimates in selected surgically fit patients with pathologically high-risk SEC, with a similar but non-significant direction for OS. This association may reflect treatment selection and differences in pathological burden rather than a causal benefit of ER before surgery. ER+CRT remains a potential organ-preserving option, but the available evidence does not establish non-inferiority to ESO. Treatment decisions should integrate detail pathological risk, surgical fitness, comorbidity, and patient preference. Prospective studies with standardized risk definitions and comparative safety outcomes are needed.

## Data Availability

The original contributions presented in the study are included in the article/**Supplementary Material**. Further inquiries can be directed to the corresponding author.

## Supplementary material

Supplementary Figure 1 Treatment ranking distributions for **(A)** overall survival, **(B)** recurrence-free survival, and **(C)** disease-specific survival. Rankings are descriptive and do not establish treatment superiority.

Supplementary Figure 2 Comparison-adjusted funnel plots for **(A)** overall survival, (B) recurrence-free survival, and **(C)** disease-specific survival.

Supplementary Figure 3 Exploratory study-level pathological-stage subgroup analyses for overall survival, recurrence-free survival, and disease-specific survival.

Supplementary Figure 4 Network sensitivity analyses. Filled squares show primary random-effects estimates, and open symbols show prespecified sensitivity scenarios.

Supplementary Figure 5 Distribution of potential effect modifiers across treatment nodes.
