# Supplementary material for: Comparative efficacy and outcomes of different treatments for pathologically high-risk superficial esophageal cancer: a systematic review and network meta-analysis
Source: Front Oncol. 2026 Jul 23;16:1866820. doi: 10.3389/fonc.2026.1866820 (PMC13441714; doi:10.3389/fonc.2026.1866820)
Supplement: Supplementary file 1 [file Table1.docx]

**Supplementary Table 1.** Database search strategies

| **Step** | **PubMed** | **Embase** | **Cochrane Library** | **Web of Science** |
| --- | --- | --- | --- | --- |
| 1 | ("Esophageal Neoplasms"[Mesh] OR esophageal cancer*[tiab] OR oesophageal cancer*[tiab] OR esophageal carcinoma*[tiab] OR cancer of esophagus[tiab]) | 'esophagus cancer'/exp | [mh "Esophageal Neoplasms"] | TS=((esophageal OR oesophageal) NEAR/2 (cancer* OR carcinoma*)) OR TS="cancer of esophagus" |
| 2 | ("Early Detection of Cancer"[Mesh] OR early stage[tiab] OR superficial[tiab] OR T1a[tiab] OR T1b[tiab] OR early esophageal[tiab]) | 'esophageal cancer*': ab, ti OR 'oesophageal cancer*': ab, ti OR 'esophageal carcinoma*': ab, ti OR 'cancer of esophagus': ab, ti | (esophageal NEXT cancer*): ti, ab, kw OR (oesophageal NEXT cancer*): ti, ab, kw OR (esophageal NEXT carcinoma*): ti, ab, kw OR "cancer of esophagus": ti, ab, kw | TS=("early stage" OR superficial OR T1a OR T1b OR (early NEAR/2 esophageal)) |
| 3 | ("Esophagectomy"[Mesh] OR esophagectomy[tiab] OR oesophagectomy[tiab] OR surgery[tiab] OR surgical[tiab] OR resection[tiab]) | 1 OR 2 | #1 OR #2 | #1 AND #2 |
| 4 | ("Endoscopic Mucosal Resection"[Mesh] OR "Endoscopic Submucosal Dissection"[Mesh] OR EMR[tiab] OR endoscopic mucosal resection[tiab] OR ESD[tiab] OR endoscopic submucosal dissection[tiab]) | 'early cancer'/exp OR 'early diagnosis'/exp | [mh "Early Detection of Cancer"] | TS=(esophagectomy OR oesophagectomy) |
| 5 | ("Chemoradiotherapy"[Mesh] OR chemoradiotherapy[tiab] OR chemoradiation[tiab] OR radiochemotherapy[tiab] OR concurrent chemoradiotherapy[tiab]) | 'early stage': ab, ti OR superficial: ab, ti OR T1a: ab, ti OR T1b: ab, ti OR 'early esophageal': ab, ti | "early stage": ti, ab, kw OR superficial: ti, ab, kw OR T1a: ti, ab, kw OR T1b: ti, ab, kw OR (early NEXT esophageal): ti, ab, kw | TS=("endoscopic mucosal resection" OR EMR OR "endoscopic submucosal dissection" OR ESD) |
| 6 | ("Chemotherapy"[Mesh] OR "Radiotherapy"[Mesh] OR chemotherapy[tiab] OR radiotherapy[tiab] OR radiation therapy[tiab]) | 4 OR 5 | #4 OR #5 | TS=(chemoradiotherapy OR chemoradiation OR radiochemotherapy OR "concurrent chemoradiotherapy") |
| 7 | #3 OR #4 OR #5 OR #6 | 'esophagectomy'/exp | [mh Esophagectomy] | TS=(chemotherapy OR radiotherapy OR "radiation therapy") |
| 8 | #1 AND #2 AND #7 | esophagectomy: ab, ti OR oesophagectomy: ab, ti OR surgery: ab, ti OR surgical: ab, ti OR resection: ab, ti | esophagectomy: ti, ab, kw OR oesophagectomy: ti, ab, kw OR surgery: ti, ab, kw OR surgical: ti, ab, kw OR resection: ti, ab, kw | #4 OR #5 OR #6 OR #7 |
| 9 |  | 7 OR 8 | #7 OR #8 | #3 AND #8 |
| 10 |  | 'endoscopic mucosal resection'/exp OR 'endoscopic submucosal dissection'/exp | [mh "Endoscopic Mucosal Resection"] OR [mh "Endoscopic Submucosal Dissection"] |  |
| 11 |  | EMR: ab, ti OR 'endoscopic mucosal resection': ab, ti OR ESD: ab, ti OR 'endoscopic submucosal dissection': ab, ti | EMR: ti, ab, kw OR "endoscopic mucosal resection": ti, ab, kw OR ESD: ti, ab, kw OR "endoscopic submucosal dissection": ti, ab, kw |  |
| 12 |  | 10 OR 11 | #10 OR #11 |  |
| 13 |  | 'chemoradiotherapy'/exp | [mh Chemoradiotherapy] |  |
| 14 |  | chemoradiotherapy: ab, ti OR chemoradiation: ab, ti OR radiochemotherapy: ab, ti OR 'concurrent chemoradiotherapy': ab, ti | chemoradiotherapy: ti, ab, kw OR chemoradiation: ti, ab, kw OR radiochemotherapy: ti, ab, kw OR "concurrent chemoradiotherapy": ti, ab, kw |  |
| 15 |  | 13 OR 14 | #13 OR #14 |  |
| 16 |  | 'chemotherapy'/exp OR 'radiotherapy'/exp | [mh Chemotherapy] OR [mh Radiotherapy] |  |
| 17 |  | chemotherapy: ab, ti OR radiotherapy: ab, ti OR 'radiation therapy': ab, ti | chemotherapy: ti, ab, kw OR radiotherapy: ti, ab, kw OR "radiation therapy": ti, ab, kw |  |
| 18 |  | 16 OR 17 | #16 OR #17 |  |
| 19 |  | 9 OR 12 OR 15 OR 18 | #9 OR #12 OR #15 OR #18 |  |
| 20 |  | 3 AND 6 AND 19 | #3 AND #6 AND #19 |  |
| 21 |  |  | random*: ti, ab, kw OR placebo*: ti, ab, kw OR [mh "Randomized Controlled Trials"] OR [mh "Double-Blind Method"] OR [mh "Single-Blind Method"] OR trial: ti OR groups: ti, ab, kw |  |
| 22 |  |  | #20 AND #21 |  |

*Note.* Databases were searched from inception through December 17, 2025.
