# Supplementary material for: Comparative efficacy and outcomes of different treatments for pathologically high-risk superficial esophageal cancer: a systematic review and network meta-analysis
Source: Front Oncol. 2026 Jul 23;16:1866820. doi: 10.3389/fonc.2026.1866820 (PMC13441714; doi:10.3389/fonc.2026.1866820)
Supplement: Supplementary file 2 [file Table10.docx]

**Supplementary Table 10.** Network-wide sensitivity and model-form analyses with corresponding diagnostics

| **Outcome** | **Analysis** | **Treatment** | **Reference** | **Studies** | **HR [95% CI]** | **P value** | **τ²** | **I²** | **P between designs** |
| --- | --- | --- | --- | --- | --- | --- | --- | --- | --- |
| OS | Excluding ER+SAT | ER+ESO | ESO | 28 | 0.57 [0.31; 1.06] | 0.074 | 0.175 | 20.9% | 0.152 |
| OS | Excluding ER+SAT | ER+CRT | ESO | 28 | 0.91 [0.45; 1.83] | 0.791 | — | — | — |
| OS | Excluding ER+SAT | ER | ESO | 28 | 0.87 [0.48; 1.58] | 0.656 | — | — | — |
| OS | Excluding ER+SAT | ER+RT | ESO | 28 | 1.20 [0.50; 2.87] | 0.678 | — | — | — |
| OS | Excluding ER+RT | ER+ESO | ESO | 31 | 0.61 [0.35; 1.06] | 0.079 | 0.073 | 8.9% | 0.245 |
| OS | Excluding ER+RT | ER+CRT | ESO | 31 | 0.95 [0.50; 1.80] | 0.878 | — | — | — |
| OS | Excluding ER+RT | ER | ESO | 31 | 0.86 [0.51; 1.45] | 0.574 | — | — | — |
| OS | Excluding ER+RT | ER+SAT | ESO | 31 | 0.89 [0.37; 2.14] | 0.792 | — | — | — |
| OS | Verified propensity-score–matched effect cohorts | ER+ESO | ESO | 7 | 0.75 [0.36; 1.57] | 0.443 | 0.014 | 28.1% | 0.082 |
| OS | Verified propensity-score–matched effect cohorts | ER+CRT | ESO | 7 | 0.30 [0.05; 1.85] | 0.193 | — | — | — |
| OS | Verified propensity-score–matched effect cohorts | ER | ESO | 7 | 0.47 [0.06; 3.81] | 0.478 | — | — | — |
| OS | Verified propensity-score–matched effect cohorts | ER+RT | ESO | 7 | 0.36 [0.05; 2.68] | 0.320 | — | — | — |
| OS | Post-ER strategies only | ER+ESO | ER | 25 | 0.48 [0.26; 0.87] | 0.016 | 0.136 | 18.7% | 0.280 |
| OS | Post-ER strategies only | ER+CRT | ER | 25 | 0.82 [0.45; 1.49] | 0.507 | — | — | — |
| OS | Post-ER strategies only | ER+SAT | ER | 25 | 1.00 [0.47; 2.14] | 0.995 | — | — | — |
| OS | Post-ER strategies only | ER+RT | ER | 25 | 1.12 [0.52; 2.40] | 0.773 | — | — | — |
| OS | Common-effect model | ER+ESO | ESO | 34 | 0.58 [0.35; 0.96] | 0.033 | 0 (assumed) | NA | NA |
| OS | Common-effect model | ER+CRT | ESO | 34 | 0.87 [0.50; 1.53] | 0.638 | — | — | — |
| OS | Common-effect model | ER | ESO | 34 | 0.90 [0.57; 1.42] | 0.660 | — | — | — |
| OS | Common-effect model | ER+SAT | ESO | 34 | 0.94 [0.42; 2.09] | 0.879 | — | — | — |
| OS | Common-effect model | ER+RT | ESO | 34 | 1.34 [0.69; 2.61] | 0.386 | — | — | — |
| RFS | Excluding ER+SAT | ER+ESO | ESO | 22 | 0.50 [0.24; 1.02] | 0.056 | 0.324 | 54.1% | <0.001 |
| RFS | Excluding ER+SAT | ER+CRT | ESO | 22 | 0.55 [0.24; 1.22] | 0.142 | — | — | — |
| RFS | Excluding ER+SAT | ER | ESO | 22 | 0.64 [0.33; 1.24] | 0.183 | — | — | — |
| RFS | Excluding ER+SAT | ER+RT | ESO | 22 | 0.75 [0.24; 2.30] | 0.616 | — | — | — |
| RFS | Excluding ER+RT | ER+ESO | ESO | 25 | 0.55 [0.29; 1.07] | 0.078 | 0.208 | 45.8% | 0.002 |
| RFS | Excluding ER+RT | ER+CRT | ESO | 25 | 0.61 [0.29; 1.29] | 0.198 | — | — | — |
| RFS | Excluding ER+RT | ER | ESO | 25 | 0.61 [0.34; 1.11] | 0.108 | — | — | — |
| RFS | Excluding ER+RT | ER+SAT | ESO | 25 | 0.54 [0.16; 1.76] | 0.303 | — | — | — |
| RFS | Verified propensity-score–matched effect cohorts | ER+ESO | ESO | 5 | 0.64 [0.27; 1.52] | 0.314 | <0.001 | 0.0% | NE |
| RFS | Verified propensity-score–matched effect cohorts | ER+CRT | ESO | 5 | 2.70 [0.46; 15.87] | 0.272 | — | — | — |
| RFS | Verified propensity-score–matched effect cohorts | ER | ESO | 5 | 0.07 [0.00; 1.53] | 0.090 | — | — | — |
| RFS | Verified propensity-score–matched effect cohorts | ER+RT | ESO | 5 | 0.03 [0.00; 0.83] | 0.038 | — | — | — |
| RFS | Post-ER strategies only | ER+ESO | ER | 19 | 0.55 [0.24; 1.29] | 0.172 | 0.615 | 63.0% | <0.001 |
| RFS | Post-ER strategies only | ER+CRT | ER | 19 | 0.66 [0.27; 1.62] | 0.364 | — | — | — |
| RFS | Post-ER strategies only | ER+SAT | ER | 19 | 0.86 [0.25; 2.96] | 0.817 | — | — | — |
| RFS | Post-ER strategies only | ER+RT | ER | 19 | 0.92 [0.26; 3.28] | 0.898 | — | — | — |
| RFS | Common-effect model | ER+ESO | ESO | 26 | 0.66 [0.40; 1.09] | 0.103 | 0 (assumed) | NA | NA |
| RFS | Common-effect model | ER+CRT | ESO | 26 | 0.65 [0.37; 1.12] | 0.120 | — | — | — |
| RFS | Common-effect model | ER | ESO | 26 | 0.60 [0.39; 0.94] | 0.024 | — | — | — |
| RFS | Common-effect model | ER+SAT | ESO | 26 | 0.52 [0.19; 1.44] | 0.210 | — | — | — |
| RFS | Common-effect model | ER+RT | ESO | 26 | 1.08 [0.54; 2.14] | 0.828 | — | — | — |
| DSS | Excluding ER+SAT | ER+ESO | ESO | 11 | 0.20 [0.06; 0.62] | 0.006 | 0.043 | 0.0% | 0.294 |
| DSS | Excluding ER+SAT | ER+CRT | ESO | 11 | 0.72 [0.19; 2.73] | 0.626 | — | — | — |
| DSS | Excluding ER+SAT | ER | ESO | 11 | 0.50 [0.22; 1.14] | 0.098 | — | — | — |
| DSS | Excluding ER+SAT | ER+RT | ESO | 11 | 1.19 [0.14; 10.37] | 0.874 | — | — | — |
| DSS | Excluding ER+RT | ER+ESO | ESO | 15 | 0.21 [0.07; 0.62] | 0.005 | <0.001 | 0.0% | 0.428 |
| DSS | Excluding ER+RT | ER+CRT | ESO | 15 | 0.72 [0.20; 2.60] | 0.614 | — | — | — |
| DSS | Excluding ER+RT | ER | ESO | 15 | 0.50 [0.23; 1.09] | 0.082 | — | — | — |
| DSS | Excluding ER+RT | ER+SAT | ESO | 15 | 1.03 [0.25; 4.29] | 0.966 | — | — | — |
| DSS | Outcome-specific cohorts only | ER+ESO | ESO | 15 | 0.19 [0.06; 0.64] | 0.007 | 0.045 | 0.0% | 0.429 |
| DSS | Outcome-specific cohorts only | ER+CRT | ESO | 15 | 0.69 [0.17; 2.78] | 0.601 | — | — | — |
| DSS | Outcome-specific cohorts only | ER | ESO | 15 | 0.47 [0.18; 1.19] | 0.112 | — | — | — |
| DSS | Outcome-specific cohorts only | ER+SAT | ESO | 15 | 0.96 [0.21; 4.44] | 0.961 | — | — | — |
| DSS | Outcome-specific cohorts only | ER+RT | ESO | 15 | 1.14 [0.13; 10.37] | 0.905 | — | — | — |
| DSS | Verified propensity-score–matched effect cohorts | NE | ESO | 2 | NE | NE | NE | NE | NE |
| DSS | Post-ER strategies only | ER+ESO | ER | 12 | 0.40 [0.12; 1.29] | 0.126 | 0.275 | 0.0% | 0.281 |
| DSS | Post-ER strategies only | ER+CRT | ER | 12 | 1.57 [0.42; 5.90] | 0.507 | — | — | — |
| DSS | Post-ER strategies only | ER+SAT | ER | 12 | 2.00 [0.53; 7.54] | 0.306 | — | — | — |
| DSS | Post-ER strategies only | ER+RT | ER | 12 | 2.60 [0.25; 27.42] | 0.427 | — | — | — |
| DSS | Common-effect model | ER+ESO | ESO | 16 | 0.21 [0.07; 0.62] | 0.005 | 0 (assumed) | NA | NA |
| DSS | Common-effect model | ER+CRT | ESO | 16 | 0.72 [0.20; 2.60] | 0.614 | — | — | — |
| DSS | Common-effect model | ER | ESO | 16 | 0.50 [0.23; 1.09] | 0.082 | — | — | — |
| DSS | Common-effect model | ER+SAT | ESO | 16 | 1.03 [0.25; 4.29] | 0.966 | — | — | — |
| DSS | Common-effect model | ER+RT | ESO | 16 | 1.19 [0.15; 9.68] | 0.869 | — | — | — |

*Note.* Matched-cohort analyses include outcome-specific propensity-score–matched cohorts; the corresponding DSS network was disconnected. The post-ER analysis uses ER as the reference and addresses a different estimand. The common-effect model assumes τ²=0. DSS, disease-specific survival; HR, hazard ratio; NA, not applicable; NE, not estimable; OS, overall survival; PSM, propensity-score matched; RFS, recurrence-free survival.
