# Supplementary material for: Comparative efficacy and outcomes of different treatments for pathologically high-risk superficial esophageal cancer: a systematic review and network meta-analysis
Source: Front Oncol. 2026 Jul 23;16:1866820. doi: 10.3389/fonc.2026.1866820 (PMC13441714; doi:10.3389/fonc.2026.1866820)
Supplement: Supplementary file 3 [file Table11.docx]

**Supplementary Table 11.** Design-detachment analysis of recurrence-free survival inconsistency

| **Detached design** | **Q after detachment** | **df** | **P after detachment** | **Overall P between designs** | **Influential design** | **Interpretation** |
| --- | --- | --- | --- | --- | --- | --- |
| ER:ER+ESO | 9.01 | 8 | 0.342 | <0.001 | Yes | Global inconsistency was no longer statistically significant |
| ER:ER+CRT:ER+ESO | 18.04 | 7 | 0.012 | <0.001 | No | Global inconsistency remained statistically significant |
| ER+CRT:ER+ESO:ER+RT | 22.99 | 7 | 0.002 | <0.001 | No | Global inconsistency remained statistically significant |
| ER:ER+RT | 23.18 | 8 | 0.003 | <0.001 | No | Global inconsistency remained statistically significant |
| ER+CRT:ER+ESO | 28.01 | 8 | <0.001 | <0.001 | No | Global inconsistency remained statistically significant |
| ER:ER+SAT | 28.70 | 8 | <0.001 | <0.001 | No | Global inconsistency remained statistically significant |
| ER+ESO:ER+SAT | 28.70 | 8 | <0.001 | <0.001 | No | Global inconsistency remained statistically significant |
| ESO:ER+CRT:ER+ESO | 30.28 | 7 | <0.001 | <0.001 | No | Global inconsistency remained statistically significant |
| ER:ER+CRT | 30.31 | 8 | <0.001 | <0.001 | No | Global inconsistency remained statistically significant |
| ESO:ER | 30.41 | 8 | <0.001 | <0.001 | No | Global inconsistency remained statistically significant |
| ESO:ER+ESO | 30.42 | 8 | <0.001 | <0.001 | No | Global inconsistency remained statistically significant |

*Note.* An influential design was defined as one whose detachment changed the global between-design test to P ≥ 0.05. No study was excluded on the basis of this diagnostic analysis. df, degrees of freedom; RFS, recurrence-free survival.
