# Supplementary material for: Comparative efficacy and outcomes of different treatments for pathologically high-risk superficial esophageal cancer: a systematic review and network meta-analysis
Source: Front Oncol. 2026 Jul 23;16:1866820. doi: 10.3389/fonc.2026.1866820 (PMC13441714; doi:10.3389/fonc.2026.1866820)
Supplement: Supplementary file 4 [file Table2.docx]

**Supplementary Table 2.** General characteristics and treatment-node definitions of the included studies

| **No.** | **Study** | **Design** | **Country/region** | **Recruitment period** | **Follow-up, months** | **Network treatments and ER+SAT components** |
| --- | --- | --- | --- | --- | --- | --- |
| 1 | An et al., 2025 | Retrospective cohort | China | January 2011 to January 2021 | ER: 38; ESO: 35 | ER / ESO |
| 2 | Beaufort et al., 2024 | Retrospective cohort | Netherlands | January 2012 to December 2017 | 66 | ER / ER+SAT; ER+SAT: ESO (10), CRT (4), selective lymph-node dissection (1) |
| 3 | Dermine et al., 2020 | Retrospective cohort | France | 2015 to 2020 | ER: 19; ER+CRT: 28 | ER+CRT / ER |
| 4 | Emi et al., 2022 | Retrospective cohort | Japan | January 2000 to June 2017 | 60 | ER+ESO / ER+CRT |
| 5 | Ikeda et al., 2015 | Retrospective cohort | Japan | January 2005 to December 2010 | 36 | ER+ESO / ER+CRT / ER |
| 6 | Kadota et al., 2022 | Retrospective cohort | Japan | January 2009 to December 2017 | 60.6 | ER / ER+ESO / ER+CRT |
| 7 | Kanie et al., 2021 | Retrospective cohort | Japan | 2005 to 2019 | 50 | ER+CRT / ER+ESO |
| 8 | Katada et al., 2023 | Retrospective cohort | Japan | August 1992 to April 2016 | 58.6 | ER+CRT / ER+ESO |
| 9 | Kim et al., 2023 | Retrospective cohort | Korea | January 2003 to December 2019 | 50.04 | ESO / ER+ESO |
| 10 | Koterazawa et al., 2018 | Retrospective cohort | Japan | 2005 to 2016 | ER+ESO: 45; ER+CRT: 41 | ER+ESO / ER+CRT |
| 11 | Kubo et al., 2025 | Retrospective cohort | Japan | August 2007 to December 2020 | 82 | ER+CRT / ER+ESO |
| 12 | Kudou et al., 2017 | Retrospective cohort | Japan | January 2008 to December 2015 | NR | ER+ESO / ER+SAT; ER+SAT: chemotherapy (2), CRT (7), observation (12) |
| 13 | Lee et al., 2020 | Retrospective cohort | Korea | January 2011 to December 2017 | ER: 33; ESO: 34 | ER / ESO |
| 14 | Lee et al., 2018 | Retrospective cohort | Korea | June 1999 to June 2014 | 43 | ER / ER+SAT; ER+SAT: RT (2), CRT (5), ESO (7) |
| 15 | Li et al., 2023 | Retrospective cohort | China | January 2012 to July 2019 | 57.8 | ER / ER+ESO / ER+CRT |
| 16 | Lin et al., 2025 | Retrospective cohort | China | 2000 to 2021 | ER+CRT: 19; ER+RT: 28 | ER+RT / ER+CRT |
| 17 | Liu et al., 2023 | Retrospective cohort | China | January 2010 to December 2018 | ER: 43; ESO: 35 | ER / ESO |
| 18 | Minamide et al., 2023 | Retrospective cohort | Japan | June 2009 to June 2020 | 49.1 | ER / ER+SAT; ER+SAT: ESO (3), CRT (7) |
| 19 | Miyata et al., 2021 | Retrospective cohort | Japan | January 2006 to August 2018 | 60.3 | ER+ESO / ER+CRT |
| 20 | Naito et al., 2022 | Retrospective cohort | Japan | April 2005 to December 2016 | NR | ER+CRT / ER+ESO |
| 21 | Nakajo et al., 2019 | Retrospective cohort | Japan | January 2010 to July 2014 | ER+SAT: 49; ER: 40 | ER+SAT / ER; ER+SAT: CRT (26), ESO (4), RT (2) |
| 22 | Nishibuchi et al., 2020 | Retrospective cohort | Japan | 2000 to 2014 | 74 | ER+CRT / ER+RT |
| 23 | Plum et al., 2018 | Retrospective cohort | Germany | 2000 to 2015 | 66 | ER+ESO / ESO |
| 24 | Song et al., 2021 | Retrospective cohort | Korea | January 2008 to December 2016 | ER: 34.4; ER+ESO: 41.7 | ER / ER+ESO |
| 25 | Suzuki et al., 2022 | Retrospective cohort | Japan | January 2008 to December 2021 | ER+CRT: 46; ER+ESO: 56 | ER+CRT / ER+ESO |
| 26 | Takeuchi et al., 2018 | Retrospective cohort | Japan | July 2008 to June 2014 | NR | ESO / ER+ESO / ER+CRT |
| 27 | Takahashi et al., 2018 | Retrospective cohort | Japan | August 2004 to November 2013 | 71.5 | ER+SAT / ER; ER+SAT: CRT (9), ESO (2), ESO plus chemotherapy (1) |
| 28 | Tanaka et al., 2024 | Retrospective cohort | Japan | 2006 to August 2017 | ER+ESO: 63; ER+CRT: 53; ER+RT: 44 | ER+ESO / ER+CRT / ER+RT |
| 29 | Tanaka et al., 2019 | Retrospective cohort | Japan | 2002 to 2013 | NR | ER+ESO / ER+CRT |
| 30 | Wang et al., 2022 | Retrospective cohort | Taiwan, China | January 2008 to December 2016 | ER: 49.2; ESO: 50.9 | ER / ESO |
| 31 | Wang et al., 2018 | Retrospective cohort | China | March 2011 to March 2015 | 46 | ER+ESO / ESO |
| 32 | Xue et al., 2023 | Retrospective cohort | China | 2007 to 2018 | 38.1 | ER+ESO / ER |
| 33 | Yang et al., 2023 | Retrospective cohort | China | January 2010 to December 2019 | 53.4 | ER+RT / ER |
| 34 | Zhang et al., 2024 | Retrospective cohort | China | 2009 to 2021 | ER: 47; ESO: 63 | ER / ESO |

*Note.* ER+SAT was a heterogeneous study-defined node comprising selective surgery, chemoradiotherapy, radiotherapy, chemotherapy, lymph-node dissection, or observation. CRT, chemoradiotherapy; ER, endoscopic resection; ESO, esophagectomy; NR, not reported; RT, radiotherapy; SAT, selective adjuvant therapy.
