# Supplementary material for: Comparative efficacy and outcomes of different treatments for pathologically high-risk superficial esophageal cancer: a systematic review and network meta-analysis
Source: Front Oncol. 2026 Jul 23;16:1866820. doi: 10.3389/fonc.2026.1866820 (PMC13441714; doi:10.3389/fonc.2026.1866820)
Supplement: Supplementary file 5 [file Table3.docx]

**Supplementary Table 3.** Newcastle-Ottawa Scale assessment of the included cohort studies

| **No.** | **First author** | **Year** | **Representative exposed cohort** | **Selection of non-exposed cohort** | **Exposure ascertainment** | **Outcome absent at baseline** | **Comparability** | **Outcome assessment** | **Sufficient follow-up** | **Adequacy of follow-up** | **Total score** |
| --- | --- | --- | --- | --- | --- | --- | --- | --- | --- | --- | --- |
| 1 | An, 2025 | 2025 | 1 | 1 | 1 | 1 | 0 | 1 | 1 | 1 | 7 |
| 2 | Beaufort, 2024 | 2024 | 1 | 1 | 1 | 1 | 0 | 1 | 1 | 1 | 7 |
| 3 | Dermine, 2020 | 2020 | 1 | 1 | 1 | 1 | 0 | 1 | 0 | 0 | 5 |
| 4 | Emi, 2022 | 2022 | 1 | 1 | 1 | 1 | 0 | 1 | 1 | 0 | 6 |
| 5 | Ikeda, 2015 | 2015 | 1 | 1 | 1 | 1 | 0 | 1 | 1 | 0 | 6 |
| 6 | Kadota, 2022 | 2022 | 1 | 1 | 1 | 1 | 0 | 1 | 1 | 0 | 6 |
| 7 | Kanie, 2021 | 2021 | 1 | 1 | 1 | 1 | 0 | 1 | 1 | 0 | 6 |
| 8 | Katada, 2023 | 2023 | 1 | 1 | 1 | 1 | 0 | 1 | 1 | 0 | 6 |
| 9 | Kim, 2023 | 2023 | 1 | 1 | 1 | 1 | 2 | 1 | 1 | 0 | 8 |
| 10 | Koterazawa, 2018 | 2018 | 1 | 1 | 1 | 1 | 0 | 1 | 1 | 0 | 6 |
| 11 | Kubo, 2025 | 2025 | 1 | 1 | 1 | 1 | 2 | 1 | 1 | 0 | 8 |
| 12 | Kudou, 2017 | 2017 | 1 | 1 | 1 | 1 | 0 | 1 | 1 | 0 | 6 |
| 13 | Lee, 2020 | 2020 | 1 | 1 | 1 | 1 | 0 | 1 | 0 | 0 | 5 |
| 14 | Lee, 2018 | 2018 | 1 | 1 | 1 | 1 | 0 | 1 | 1 | 0 | 6 |
| 15 | Li, 2023 | 2023 | 1 | 1 | 1 | 1 | 0 | 1 | 1 | 0 | 6 |
| 16 | Lin, 2025 | 2025 | 1 | 1 | 1 | 1 | 2 | 1 | 0 | 0 | 7 |
| 17 | Liu, 2023 | 2023 | 1 | 1 | 1 | 1 | 2 | 1 | 1 | 0 | 8 |
| 18 | Minamide, 2023 | 2023 | 1 | 1 | 1 | 1 | 0 | 1 | 1 | 0 | 6 |
| 19 | Miyata, 2021 | 2021 | 1 | 1 | 1 | 1 | 0 | 1 | 1 | 0 | 6 |
| 20 | Naito, 2022 | 2022 | 1 | 1 | 1 | 1 | 2 | 1 | 1 | 0 | 8 |
| 21 | Nakajo, 2019 | 2019 | 1 | 1 | 1 | 1 | 2 | 1 | 1 | 0 | 8 |
| 22 | Nishibuchi, 2020 | 2020 | 1 | 1 | 1 | 1 | 0 | 1 | 1 | 1 | 7 |
| 23 | Plum, 2018 | 2018 | 1 | 1 | 1 | 1 | 2 | 1 | 1 | 0 | 8 |
| 24 | Song, 2021 | 2021 | 1 | 1 | 1 | 1 | 2 | 1 | 1 | 0 | 8 |
| 25 | Suzuki, 2022 | 2022 | 1 | 1 | 1 | 1 | 0 | 1 | 1 | 0 | 6 |
| 26 | Takeuchi, 2018 | 2018 | 1 | 1 | 1 | 1 | 0 | 1 | 1 | 0 | 6 |
| 27 | Takahashi, 2018 | 2018 | 1 | 1 | 1 | 1 | 0 | 1 | 1 | 1 | 7 |
| 28 | Tanaka, 2024 | 2024 | 1 | 1 | 1 | 1 | 0 | 1 | 1 | 0 | 6 |
| 29 | Tanaka, 2019 | 2019 | 1 | 1 | 1 | 1 | 0 | 1 | 1 | 0 | 6 |
| 30 | Wang, 2022 | 2022 | 1 | 1 | 1 | 1 | 0 | 1 | 1 | 0 | 6 |
| 31 | Wang, 2018 | 2018 | 1 | 1 | 1 | 1 | 2 | 1 | 1 | 1 | 9 |
| 32 | Xue, 2023 | 2023 | 1 | 1 | 1 | 1 | 0 | 1 | 1 | 0 | 6 |
| 33 | Yang, 2023 | 2023 | 1 | 1 | 1 | 1 | 2 | 1 | 1 | 0 | 8 |
| 34 | Zhang, 2024 | 2024 | 1 | 1 | 1 | 1 | 2 | 1 | 1 | 0 | 8 |

*Note.* Selection and outcome items are scored 0 or 1; comparability is scored 0–2. The maximum total score is 9, with higher scores indicating lower risk of bias. NOS, Newcastle-Ottawa Scale.
