# Supplementary material for: Comparative efficacy and outcomes of different treatments for pathologically high-risk superficial esophageal cancer: a systematic review and network meta-analysis
Source: Front Oncol. 2026 Jul 23;16:1866820. doi: 10.3389/fonc.2026.1866820 (PMC13441714; doi:10.3389/fonc.2026.1866820)
Supplement: Supplementary file 6 [file Table4.docx]

**Supplementary Table 4.** Detailed distribution of pathological high-risk features across treatment arms

| **Study** | **Treatment** | **Network N** | **Pathology N** | **Ly, n/N (%)** | **V, n/N (%)** | **pT1a+LVI, n/N (%)** | **pT1b+LVI, n/N (%)** | **Positive VM, n/N (%)** | **Poor differentiation, n/N (%)** |
| --- | --- | --- | --- | --- | --- | --- | --- | --- | --- |
| An et al., 2025 | ER | 95 | 95 | NR | NR | 1/59 (1.7%) | 2/36 (5.6%) | NR | 95/95 (100.0%) |
| An et al., 2025 | ESO | 86 | 86 | NR | NR | NR | NR | NR | 86/86 (100.0%) |
| Beaufort et al., 2024 | ER | 17 | 17 | NR | NR | NR | NR | NR | NR |
| Beaufort et al., 2024 | ER+SAT | 15 | 15 | NR | NR | NR | NR | NR | NR |
| Dermine et al., 2020 | ER+CRT | 28 | 28 | NR | NR | NR | NR | 0/28 (0.0%) | 5/28 (17.9%) |
| Dermine et al., 2020 | ER | 13 | 13 | NR | NR | 1/8 (12.5%) | 2/5 (40.0%) | 0/13 (0.0%) | 5/13 (38.5%) |
| Emi et al., 2022 | ER+ESO | 30 | 30 | 11/30 (36.7%) | 5/30 (16.7%) | NR | NR | 5/30 (16.7%) | NR |
| Emi et al., 2022 | ER+CRT | 37 | 37 | 15/37 (40.5%) | 7/37 (18.9%) | NR | NR | 8/37 (21.6%) | NR |
| Ikeda et al., 2015 | ER+ESO | 15 | 15 | NR | NR | NR | 6/15 (40.0%) | 3/15 (20.0%) | 1/15 (6.7%) |
| Ikeda et al., 2015 | ER+CRT | 11 | 11 | NR | NR | NR | 8/11 (72.7%) | 4/11 (36.4%) | 1/11 (9.1%) |
| Ikeda et al., 2015 | ER | 17 | 17 | NR | NR | NR | 3/17 (17.6%) | 2/17 (11.8%) | 1/17 (5.9%) |
| Kadota et al., 2022 | ER | 21 | 21 | 5/21 (23.8%) | 5/21 (23.8%) | 2/2 (100.0%) | 5/19 (26.3%) | 0/21 (0.0%) | NR |
| Kadota et al., 2022 | ER+ESO | 18 | 18 | 4/18 (22.2%) | 9/18 (50.0%) | 2/2 (100.0%) | 10/16 (62.5%) | 0/18 (0.0%) | NR |
| Kadota et al., 2022 | ER+CRT | 50 | 50 | 18/50 (36.0%) | 16/50 (32.0%) | 10/10 (100.0%) | 19/40 (47.5%) | 0/50 (0.0%) | NR |
| Kanie et al., 2021 | ER+CRT | 52 | 52 | 23/52 (44.2%) | 9/52 (17.3%) | 19/19 (100.0%) | NR | 6/52 (11.5%) | NR |
| Kanie et al., 2021 | ER+ESO | 56 | 56 | 38/56 (67.9%) | 11/56 (19.6%) | 18/18 (100.0%) | NR | 11/56 (19.6%) | NR |
| Katada et al., 2023 | ER+CRT | 131 | 131 | NR | NR | 60/60 (100.0%) | NR | 0/131 (0.0%) | NR |
| Katada et al., 2023 | ER+ESO | 58 | 58 | NR | NR | 29/29 (100.0%) | NR | 0/58 (0.0%) | NR |
| Kim et al., 2023 | ESO | 34 | 34 | NR | NR | NR | NR | NR | 1/34 (2.9%) |
| Kim et al., 2023 | ER+ESO | 34 | 34 | NR | NR | NR | NR | NR | 0/34 (0.0%) |
| Koterazawa et al., 2018 | ER+ESO | 28 | 28 | 16/28 (57.1%) | 1/28 (3.6%) | 7/8 (87.5%) | NR | 3/28 (10.7%) | NR |
| Koterazawa et al., 2018 | ER+CRT | 31 | 31 | 11/31 (35.5%) | 3/31 (9.7%) | NR | NR | 4/31 (12.9%) | NR |
| Kubo et al., 2025 | ER+CRT | 29 | 29 | 19/29 (65.5%) | 8/29 (27.6%) | NR | NR | 1/29 (3.4%) | NR |
| Kubo et al., 2025 | ER+ESO | 29 | 29 | 13/29 (44.8%) | 8/29 (27.6%) | NR | NR | 1/29 (3.4%) | NR |
| Kudou et al., 2017 | ER+ESO | 16 | 16 | 10/16 (62.5%) | 4/16 (25.0%) | 5/9 (55.6%) | 5/7 (71.4%) | 2/16 (12.5%) | NR |
| Kudou et al., 2017 | ER+SAT | 21 | 21 | 8/21 (38.1%) | 4/21 (19.0%) | NR | NR | 4/21 (19.0%) | NR |
| Lee et al., 2020 | ER | 8 | 8 | NR | NR | NR | NR | NR | NR |
| Lee et al., 2020 | ESO | 46 | 46 | NR | NR | NR | NR | NR | NR |
| Lee et al., 2018 | ER | 10 | 10 | NR | NR | 2/2 (100.0%) | 0/8 (0.0%) | 0/10 (0.0%) | NR |
| Lee et al., 2018 | ER+SAT | 14 | 14 | NR | NR | 1/1 (100.0%) | 1/13 (7.7%) | 0/14 (0.0%) | NR |
| Li et al., 2023 | ER | 22 | 22 | NR | NR | NR | NR | 6/22 (27.3%) | NR |
| Li et al., 2023 | ER+ESO | 23 | 23 | NR | NR | NR | NR | NR | NR |
| Li et al., 2023 | ER+CRT | 7 | 7 | NR | NR | NR | NR | NR | NR |
| Lin et al., 2025 | ER+RT | 13 | 13 | NR | NR | NR | NR | NR | 4/13 (30.8%) |
| Lin et al., 2025 | ER+CRT | 13 | 13 | NR | NR | NR | NR | NR | 3/13 (23.1%) |
| Liu et al., 2023 | ER | 19 | 19 | NR | 2/19 (10.5%) | NR | NR | NR | NR |
| Liu et al., 2023 | ESO | 19 | 19 | NR | 3/19 (15.8%) | NR | NR | NR | NR |
| Minamide et al., 2023 | ER | 11 | 11 | 4/11 (36.4%) | 3/11 (27.3%) | NR | NR | 2/11 (18.2%) | NR |
| Minamide et al., 2023 | ER+SAT | 10 | 10 | 3/10 (30.0%) | 3/10 (30.0%) | NR | NR | 2/10 (20.0%) | NR |
| Miyata et al., 2021 | ER+ESO | 37 | 37 | 18/37 (48.6%) | 6/37 (16.2%) | 11/11 (100.0%) | 11/26 (42.3%) | 2/37 (5.4%) | NR |
| Miyata et al., 2021 | ER+CRT | 123 | 123 | 53/123 (43.1%) | 23/123 (18.7%) | 37/37 (100.0%) | 29/86 (33.7%) | 11/123 (8.9%) | NR |
| Naito et al., 2022 | ER+CRT | 33 | 33 | NR | NR | NR | NR | NR | NR |
| Naito et al., 2022 | ER+ESO | 29 | 29 | NR | NR | NR | NR | NR | NR |
| Nakajo et al., 2019 | ER+SAT | 32 | 32 | 15/32 (46.9%) | 11/32 (34.4%) | NR | NR | 9/32 (28.1%) | NR |
| Nakajo et al., 2019 | ER | 41 | 41 | 11/41 (26.8%) | 14/41 (34.1%) | NR | NR | 4/41 (9.8%) | NR |
| Nishibuchi et al., 2020 | ER+CRT | 24 | NR | NR | NR | NR | NR | NR | NR |
| Nishibuchi et al., 2020 | ER+RT | 13 | NR | NR | NR | NR | NR | NR | NR |
| Plum et al., 2018 | ER+ESO | 37 | 37 | NR | NR | NR | NR | NR | NR |
| Plum et al., 2018 | ESO | 37 | 37 | NR | NR | NR | NR | NR | NR |
| Song et al., 2021 | ER | 23 | 23 | NR | NR | 6/6 (100.0%) | 2/17 (11.8%) | 0/23 (0.0%) | 1/23 (4.3%) |
| Song et al., 2021 | ER+ESO | 29 | 29 | NR | NR | 1/1 (100.0%) | 11/28 (39.3%) | 0/29 (0.0%) | 1/29 (3.4%) |
| Suzuki et al., 2022 | ER+CRT | 34 | 34 | 9/34 (26.5%) | 12/34 (35.3%) | NR | NR | 7/34 (20.6%) | NR |
| Suzuki et al., 2022 | ER+ESO | 26 | 26 | 13/26 (50.0%) | 8/26 (30.8%) | NR | NR | 4/26 (15.4%) | NR |
| Takahashi et al., 2018 | ER+SAT | 12 | 12 | NR | NR | NR | NR | 0/12 (0.0%) | NR |
| Takahashi et al., 2018 | ER | 11 | 11 | NR | NR | NR | NR | 0/11 (0.0%) | NR |
| Takeuchi et al., 2018 | ESO | 51 | NR | NR | NR | NR | NR | NR | NR |
| Takeuchi et al., 2018 | ER+ESO | 19 | 19 | NR | NR | NR | NR | NR | NR |
| Takeuchi et al., 2018 | ER+CRT | 13 | NR | NR | NR | NR | NR | NR | NR |
| Tanaka et al., 2024 | ER+ESO | 57 | 57 | 36/57 (63.2%) | 22/57 (38.6%) | NR | NR | 4/57 (7.0%) | NR |
| Tanaka et al., 2024 | ER+CRT | 125 | 125 | 59/125 (47.2%) | 41/125 (32.8%) | NR | NR | 15/125 (12.0%) | NR |
| Tanaka et al., 2024 | ER+RT | 38 | 38 | 19/38 (50.0%) | 27/38 (71.1%) | NR | NR | 4/38 (10.5%) | NR |
| Tanaka et al., 2019 | ER+ESO | 19 | 19 | 12/19 (63.2%) | 15/19 (78.9%) | NR | 17/19 (89.5%) | 2/19 (10.5%) | NR |
| Tanaka et al., 2019 | ER+CRT | 33 | 33 | 13/33 (39.4%) | 9/33 (27.3%) | NR | 16/33 (48.5%) | 1/33 (3.0%) | NR |
| Wang et al., 2022 | ER | 21 | 21 | NR | NR | NR | 2/21 (9.5%) | 3/21 (14.3%) | NR |
| Wang et al., 2022 | ESO | 28 | 28 | NR | NR | NR | NR | NR | NR |
| Wang et al., 2018 | ER+ESO | 48 | 48 | NR | NR | 0/24 (0.0%) | 0/24 (0.0%) | 1/48 (2.1%) | 8/48 (16.7%) |
| Wang et al., 2018 | ESO | 96 | 96 | NR | NR | NR | NR | NR | 19/96 (19.8%) |
| Xue et al., 2023 | ER+ESO | 42 | 42 | NR | NR | NR | 13/42 (31.0%) | 10/42 (23.8%) | 12/42 (28.6%) |
| Xue et al., 2023 | ER | 124 | 124 | NR | NR | NR | 16/124 (12.9%) | 3/124 (2.4%) | 52/124 (41.9%) |
| Yang et al., 2023 | ER+RT | 36 | 36 | NR | NR | NR | 12/36 (33.3%) | 3/36 (8.3%) | 8/36 (22.2%) |
| Yang et al., 2023 | ER | 36 | 36 | NR | NR | NR | 9/36 (25.0%) | 3/36 (8.3%) | 6/36 (16.7%) |
| Zhang et al., 2024 | ER | 47 | 47 | NR | NR | NR | 4/47 (8.5%) | NR | 8/47 (17.0%) |
| Zhang et al., 2024 | ESO | 73 | 73 | NR | NR | NR | 5/73 (6.8%) | NR | 20/73 (27.4%) |

*Note.* This table supplements Table 1 with high-risk pathological features not reported there in detail. For pT1a+LVI and pT1b+LVI, denominators are the corresponding pT1a and pT1b populations; other pathological proportions use the pathology cohort denominator. ER, endoscopic resection; LVI, lymphovascular invasion; Ly, lymphatic invasion; NR, not reported; V, venous invasion; VM, vertical margin.
