# Supplementary material for: Comparative efficacy and outcomes of different treatments for pathologically high-risk superficial esophageal cancer: a systematic review and network meta-analysis
Source: Front Oncol. 2026 Jul 23;16:1866820. doi: 10.3389/fonc.2026.1866820 (PMC13441714; doi:10.3389/fonc.2026.1866820)
Supplement: Supplementary file 7 [file Table5.docx]

**Supplementary Table 5.** Distribution of baseline characteristics and potential effect modifiers across treatment nodes

| **Treatment node** | **Studies/arms** | **Total network patients** | **Reported mean age, years** | **Reported median age, years** | **Male, %** | **pT1b, %** | **LVI, %** | **Positive vertical margin, %** | **Poor differentiation, %** | **CCI/PS reporting, arms** |
| --- | --- | --- | --- | --- | --- | --- | --- | --- | --- | --- |
| ESO | 9/9 | 470 | 63.1 [59.0; 64.4] (4 arms) | 59.0 [54.0; 64.0] (2 arms) | 79.1 [57.9; 100.0] (5 arms) | 100.0 [64.7; 100.0] (7 arms) | 12.2 [6.8; 17.6] (2 arms) | NR | 27.4 [2.9; 100.0] (3 arms) | 0/9; 2/9 |
| ER+ESO | 20/20 | 650 | 62.7 [59.9; 63.1] (3 arms) | 62.5 [60.5; 68.0] (12 arms) | 86.9 [78.9; 100.0] (14 arms) | 75.9 [43.8; 100.0] (17 arms) | 41.4 [17.6; 89.5] (7 arms) | 10.6 [0.0; 23.8] (14 arms) | 5.1 [0.0; 28.6] (4 arms) | 5/20; 5/20 |
| ER+CRT | 17/17 | 774 | 66.9 [66.9; 66.9] (1 arm) | 67.5 [65.0; 69.0] (10 arms) | 88.5 [53.8; 97.0] (11 arms) | 71.3 [51.7; 100.0] (14 arms) | 53.7 [28.6; 72.7] (5 arms) | 10.2 [0.0; 36.4] (12 arms) | 17.9 [9.1; 23.1] (3 arms) | 4/17; 7/17 |
| ER | 17/17 | 536 | 66.6 [61.1; 79.6] (5 arms) | 65.0 [55.0; 77.0] (6 arms) | 77.3 [52.6; 100.0] (10 arms) | 90.5 [36.4; 100.0] (15 arms) | 21.5 [8.5; 34.8] (8 arms) | 5.4 [0.0; 27.3] (12 arms) | 17.0 [4.3; 100.0] (7 arms) | 3/17; 6/17 |
| ER+SAT | 6/6 | 104 | 71.5 [64.7; 77.3] (3 arms) | NR | 85.7 [81.2; 90.5] (3 arms) | 71.9 [41.7; 100.0] (5 arms) | 14.3 [14.3; 14.3] (1 arm) | 19.0 [0.0; 28.1] (5 arms) | NR | 1/6; 1/6 |
| ER+RT | 4/4 | 100 | NR | 73.0 [73.0; 73.0] (1 arm) | 80.6 [61.5; 94.7] (3 arms) | 100.0 [63.2; 100.0] (3 arms) | 33.3 [33.3; 33.3] (1 arm) | 9.4 [8.3; 10.5] (2 arms) | 26.5 [22.2; 30.8] (2 arms) | 1/4; 2/4 |

*Note. Values are unweighted medians [ranges] across treatment arms with arm-specific pathological data corresponding to the analyzed network cohort*. Mean and median ages are shown separately. CCI, Charlson Comorbidity Index; LVI, lymphovascular invasion; PS, performance status.
