# Supplementary material for: Comparative efficacy and outcomes of different treatments for pathologically high-risk superficial esophageal cancer: a systematic review and network meta-analysis
Source: Front Oncol. 2026 Jul 23;16:1866820. doi: 10.3389/fonc.2026.1866820 (PMC13441714; doi:10.3389/fonc.2026.1866820)
Supplement: Supplementary file 8 [file Table6.docx]

**Supplementary Table 6.** Random-effects node-splitting analysis of local inconsistency

| **Outcome** | **Comparison** | **Direct studies** | **Direct evidence** | **Network HR [95% CI]** | **Direct HR [95% CI]** | **Indirect HR [95% CI]** | **Direct/indirect ratio [95% CI]** | **P for inconsistency** |
| --- | --- | --- | --- | --- | --- | --- | --- | --- |
| Overall survival | ER vs ER+CRT | 4 | 47.2% | 1.00 [0.57; 1.76] | 0.92 [0.40; 2.08] | 1.08 [0.50; 2.35] | 0.85 [0.27; 2.62] | 0.774 |
| Overall survival | ER vs ER+ESO | 5 | 46.7% | 1.60 [0.95; 2.69] | 2.15 [1.00; 4.60] | 1.23 [0.60; 2.52] | 1.74 [0.61; 4.96] | 0.297 |
| Overall survival | ER vs ER+RT | 1 | 22.7% | 0.74 [0.36; 1.55] | 1.93 [0.41; 9.04] | 0.56 [0.24; 1.30] | 3.44 [0.59; 19.94] | 0.169 |
| Overall survival | ER vs ER+SAT | 5 | 94.6% | 0.98 [0.46; 2.10] | 1.09 [0.50; 2.38] | 0.17 [0.01; 4.36] | 6.51 [0.23; 186.04] | 0.273 |
| Overall survival | ER vs ESO | 5 | 66.3% | 0.90 [0.51; 1.58] | 0.62 [0.31; 1.25] | 1.84 [0.69; 4.89] | 0.34 [0.10; 1.13] | 0.078 |
| Overall survival | ER+CRT vs ER+ESO | 13 | 89.0% | 1.60 [1.05; 2.44] | 1.63 [1.04; 2.55] | 1.37 [0.38; 4.91] | 1.19 [0.31; 4.60] | 0.803 |
| Overall survival | ER+CRT vs ER+RT | 3 | 81.8% | 0.74 [0.41; 1.35] | 0.66 [0.34; 1.27] | 1.30 [0.32; 5.29] | 0.50 [0.11; 2.37] | 0.385 |
| Overall survival | ER+ESO vs ER+RT | 1 | 40.1% | 0.47 [0.24; 0.90] | 0.29 [0.10; 0.83] | 0.64 [0.27; 1.50] | 0.45 [0.12; 1.76] | 0.254 |
| Overall survival | ER+ESO vs ER+SAT | 1 | 7.9% | 0.62 [0.25; 1.52] | 0.11 [0.00; 2.74] | 0.71 [0.28; 1.84] | 0.15 [0.01; 4.39] | 0.273 |
| Overall survival | ER+ESO vs ESO | 4 | 58.8% | 0.56 [0.31; 1.02] | 0.88 [0.41; 1.90] | 0.30 [0.12; 0.75] | 2.95 [0.89; 9.81] | 0.078 |
| Recurrence-free survival | ER vs ER+CRT | 3 | 37.7% | 1.22 [0.61; 2.43] | 2.59 [0.84; 7.93] | 0.77 [0.32; 1.85] | 3.35 [0.81; 13.85] | 0.095 |
| Recurrence-free survival | ER vs ER+ESO | 4 | 51.2% | 1.36 [0.73; 2.51] | 1.01 [0.43; 2.39] | 1.85 [0.77; 4.47] | 0.55 [0.16; 1.87] | 0.335 |
| Recurrence-free survival | ER vs ER+RT | 1 | 38.3% | 0.88 [0.32; 2.39] | 2.29 [0.45; 11.58] | 0.48 [0.13; 1.73] | 4.74 [0.60; 37.33] | 0.139 |
| Recurrence-free survival | ER vs ER+SAT | 3 | 90.9% | 1.15 [0.39; 3.43] | 1.38 [0.44; 4.35] | 0.18 [0.00; 6.94] | 7.47 [0.17; 334.62] | 0.300 |
| Recurrence-free survival | ER vs ESO | 5 | 69.3% | 0.65 [0.34; 1.26] | 0.52 [0.24; 1.15] | 1.07 [0.33; 3.50] | 0.49 [0.12; 2.04] | 0.327 |
| Recurrence-free survival | ER+CRT vs ER+ESO | 11 | 93.2% | 1.11 [0.68; 1.83] | 1.25 [0.75; 2.08] | 0.23 [0.03; 1.55] | 5.37 [0.75; 38.31] | 0.094 |
| Recurrence-free survival | ER+CRT vs ER+RT | 1 | 60.5% | 0.72 [0.27; 1.90] | 0.48 [0.14; 1.66] | 1.36 [0.29; 6.33] | 0.35 [0.05; 2.55] | 0.302 |
| Recurrence-free survival | ER+CRT vs ESO | 1 | 11.3% | 0.54 [0.24; 1.20] | 0.64 [0.06; 6.95] | 0.52 [0.22; 1.23] | 1.22 [0.10; 15.39] | 0.879 |
| Recurrence-free survival | ER+ESO vs ER+RT | 1 | 54.3% | 0.65 [0.25; 1.69] | 0.40 [0.11; 1.46] | 1.16 [0.28; 4.79] | 0.34 [0.05; 2.36] | 0.277 |
| Recurrence-free survival | ER+ESO vs ER+SAT | 1 | 11.8% | 0.85 [0.25; 2.89] | 0.14 [0.00; 5.11] | 1.07 [0.29; 3.96] | 0.13 [0.00; 6.00] | 0.300 |
| Recurrence-free survival | ER+ESO vs ESO | 3 | 51.0% | 0.48 [0.23; 0.98] | 0.69 [0.25; 1.87] | 0.33 [0.12; 0.92] | 2.07 [0.49; 8.68] | 0.319 |
| Disease-specific survival | ER vs ER+CRT | 1 | 66.0% | 0.69 [0.24; 2.00] | 0.96 [0.26; 3.55] | 0.37 [0.06; 2.28] | 2.60 [0.28; 24.56] | 0.404 |
| Disease-specific survival | ER vs ER+ESO | 2 | 64.5% | 2.41 [0.99; 5.87] | 2.18 [0.72; 6.61] | 2.88 [0.64; 12.85] | 0.76 [0.12; 4.88] | 0.770 |
| Disease-specific survival | ER vs ER+SAT | 4 | 93.2% | 0.48 [0.15; 1.59] | 0.45 [0.13; 1.56] | 1.09 [0.01; 106.92] | 0.42 [0.00; 48.42] | 0.719 |
| Disease-specific survival | ER vs ESO | 3 | 88.8% | 0.50 [0.23; 1.09] | 0.51 [0.22; 1.17] | 0.43 [0.04; 4.54] | 1.18 [0.10; 14.48] | 0.896 |
| Disease-specific survival | ER+CRT vs ER+ESO | 5 | 75.7% | 3.48 [1.20; 10.12] | 5.71 [1.67; 19.49] | 0.75 [0.09; 6.49] | 7.66 [0.64; 92.15] | 0.109 |
| Disease-specific survival | ER+ESO vs ER+SAT | 1 | 10.4% | 0.20 [0.05; 0.85] | 0.44 [0.00; 39.31] | 0.18 [0.04; 0.85] | 2.39 [0.02; 277.22] | 0.719 |
| Disease-specific survival | ER+ESO vs ESO | 1 | 26.0% | 0.21 [0.07; 0.62] | 0.18 [0.02; 1.57] | 0.22 [0.06; 0.77] | 0.85 [0.07; 10.35] | 0.896 |

*Note.* Node splitting used the back-calculation method and a random-effects model with a common network between-study variance. HRs compare the first treatment with the second treatment. CI, confidence interval; HR, hazard ratio.
