# Supplementary material for: Comparative efficacy and outcomes of different treatments for pathologically high-risk superficial esophageal cancer: a systematic review and network meta-analysis
Source: Front Oncol. 2026 Jul 23;16:1866820. doi: 10.3389/fonc.2026.1866820 (PMC13441714; doi:10.3389/fonc.2026.1866820)
Supplement: Supplementary file 9 [file Table7.docx]

**Supplementary Table 7.** Global heterogeneity and design-by-treatment inconsistency diagnostics

| **Outcome** | **Studies** | **Comparisons** | **Treatments** | **τ²** | **I²** | **Total Q (df)** | **P** | **Within-design Q (df)** | **P** | **Between-design Q (df)** | **P** |
| --- | --- | --- | --- | --- | --- | --- | --- | --- | --- | --- | --- |
| Overall survival | 34 | 42 | 6 | 0.141 | 15.8% | 39.19 (33) | 0.212 | 27.23 (25) | 0.344 | 11.96 (8) | 0.153 |
| Recurrence-free survival | 26 | 34 | 6 | 0.321 | 51.6% | 51.70 (25) | 0.001 | 21.30 (16) | 0.167 | 30.43 (9) | <0.001 |
| Disease-specific survival | 16 | 18 | 6 | <0.001 | 0.0% | 8.48 (12) | 0.747 | 4.69 (8) | 0.790 | 3.84 (4) | 0.428 |

*Note.* Between-design Q is the design-by-treatment interaction test for global inconsistency. A two-sided P < 0.05 indicates statistical evidence of inconsistency. df, degrees of freedom.
