# Supplementary material for: Comparative efficacy and outcomes of different treatments for pathologically high-risk superficial esophageal cancer: a systematic review and network meta-analysis
Source: Front Oncol. 2026 Jul 23;16:1866820. doi: 10.3389/fonc.2026.1866820 (PMC13441714; doi:10.3389/fonc.2026.1866820)
Supplement: Supplementary file 10 [file Table8.docx]

**Supplementary Table 8.** Exploratory study-level pathological-stage subgroup analyses

| **Outcome** | **Comparison** | **Mixed pT1a/pT1b HR [95% CI]** | **Studies** | **pT1b-only HR [95% CI]** | **Studies** | **P for interaction** |
| --- | --- | --- | --- | --- | --- | --- |
| Overall survival | ER vs ESO | 0.93 [0.32; 2.72] | 21 | 0.75 [0.39; 1.45] | 10 | 0.747 |
| Overall survival | ER+CRT vs ESO | 0.65 [0.19; 2.16] | 21 | 0.92 [0.29; 2.89] | 10 | 0.676 |
| Overall survival | ER+ESO vs ESO | 0.36 [0.11; 1.15] | 21 | 0.71 [0.31; 1.62] | 10 | 0.349 |
| Overall survival | ER+RT vs ESO | 0.94 [0.22; 3.97] | 21 | 0.83 [0.24; 2.83] | 10 | 0.902 |
| Overall survival | ER+SAT vs ESO | 0.76 [0.17; 3.31] | 21 | NE | NE | NE |
| Recurrence-free survival | ER vs ESO | 0.75 [0.36; 1.57] | 16 | 0.44 [0.14; 1.40] | 8 | 0.450 |
| Recurrence-free survival | ER+CRT vs ESO | 0.30 [0.11; 0.77] | 16 | 0.84 [0.11; 6.14] | 8 | 0.354 |
| Recurrence-free survival | ER+ESO vs ESO | 0.28 [0.11; 0.70] | 16 | 0.49 [0.09; 2.74] | 8 | 0.575 |
| Recurrence-free survival | ER+RT vs ESO | 0.64 [0.22; 1.88] | 16 | 0.19 [0.02; 1.90] | 8 | 0.352 |
| Recurrence-free survival | ER+SAT vs ESO | 0.60 [0.19; 1.93] | 16 | NE | NE | NE |
| Disease-specific survival | ER vs ESO | 0.52 [0.14; 1.88] | 12 | NE | NE | NE |
| Disease-specific survival | ER+CRT vs ESO | 0.79 [0.14; 4.37] | 12 | NE | NE | NE |
| Disease-specific survival | ER+ESO vs ESO | 0.20 [0.05; 0.87] | 12 | NE | NE | NE |
| Disease-specific survival | ER+SAT vs ESO | 0.76 [0.09; 6.66] | 12 | NE | NE | NE |

*Note.* Interaction tests are exploratory. CI, confidence interval; HR, hazard ratio; NE, not estimable.
