# Supplementary material for: Comparative efficacy and outcomes of different treatments for pathologically high-risk superficial esophageal cancer: a systematic review and network meta-analysis
Source: Front Oncol. 2026 Jul 23;16:1866820. doi: 10.3389/fonc.2026.1866820 (PMC13441714; doi:10.3389/fonc.2026.1866820)
Supplement: Supplementary file 11 [file Table9.docx]

**Supplementary Table 9.** Exploratory common-slope network meta-regression

| **Outcome** | **Covariate** | **Unit** | **Analysis** | **Studies** | **Ratio of HRs [95% CI]** | **P value** |
| --- | --- | --- | --- | --- | --- | --- |
| Overall survival | T1b proportion | Per 10-percentage-point increase | Primary analysis | 32 | 0.95 [0.73; 1.24] | 0.707 |
| Recurrence-free survival | T1b proportion | Per 10-percentage-point increase | Primary analysis | 25 | 0.89 [0.64; 1.24] | 0.493 |
| Disease-specific survival | T1b proportion | Per 10-percentage-point increase | Primary analysis | 14 | 0.88 [0.48; 1.61] | 0.685 |
| Overall survival | LVI proportion | Per 10-percentage-point increase | Primary analysis | 15 | 0.73 [0.17; 3.17] | 0.673 |
| Recurrence-free survival | LVI proportion | Per 10-percentage-point increase | Primary analysis | 13 | 0.75 [0.13; 4.43] | 0.751 |
| Disease-specific survival | LVI proportion | Per 10-percentage-point increase | Primary analysis | 7 | NE | NE |

*Note.* Ratios of HRs below 1 indicate greater relative benefit at higher covariate values under the common-slope assumption. These analyses are exploratory and subject to ecological bias. CI, confidence interval; HR, hazard ratio; NE, not estimated.
